# Fresh Phytomedicines: Traditional Applications, Chemical Composition, Pharmacological Activities, Challenges, and Strategies

**DOI:** 10.3390/plants15142122

**Published:** 2026-07-09

**Authors:** Yifan Zeng, Kanglin Bai, Jianhong Xie, Xinghua Mu, Xinru Li, Yujiao Zhang, Juan Xu, Changwei Wu, Chaohai Li, Fumei He, Baozhong Duan

**Affiliations:** College of Pharmaceutical Science, Dali University, Dali 671000, China; 18759637322@163.com (Y.Z.); baikl0212@163.com (K.B.);

**Keywords:** fresh phytomedicines, traditional medicine, comparison, chemical composition, pharmacological activities

## Abstract

Fresh phytomedicines (FPs), defined as medicinal plants used in their fresh, undried state, have long been applied in traditional medical systems worldwide and represent a diverse yet underexplored source of bioactive compounds derived from classical prescriptions, ethnomedicinal practices, and medicine–food homology plants. Increasing evidence suggests that FPs possess distinct chemical and pharmacological properties compared with dried phytomedicines (DPs), although their therapeutic value has not been systematically evaluated from a modern medical perspective. This review integrates ethnomedicinal knowledge with modern pharmacological evidence to clarify the therapeutic potential of FPs, while comparing them with DPs and evaluating their potential role as context-dependent alternatives or complementary strategies that warrant further clinical investigation. A comprehensive literature search covering the period from 1956 to January 2026 was conducted across Web of Science, PubMed, Scopus, CNKI, ProQuest, and SciELO, followed by bibliometric analysis using VOSviewer 1.6.20. Available evidence indicates that FPs and DPs exhibit distinct physicochemical and pharmacological profiles. Drying enriches thermally stable constituents or leads to the formation of new compounds in DPs, whereas FPs better preserve thermosensitive and labile compounds, including polysaccharides, flavonoids, alkaloids, and volatile oils. These preserved constituents contribute to context-dependent activities such as antimicrobial, antioxidant, antidiabetic, and immunomodulatory effects. Although FPs offer unique therapeutic potential, their application is constrained by instability, limited standardization, inconsistent dosing, and insufficient clinical evidence, and future efforts should focus on improved preservation technologies, comprehensive quality standards, systematic dosage studies, and well-designed clinical trials to substantiate their clinical applicability and facilitate evidence-based integration into modern therapeutic frameworks.

## 1. Introduction

Fresh phytomedicines (FPs) are medicinal plants used immediately after harvest and have long been integral to traditional healing systems for thousands of years. Historical records consistently highlight the perceived therapeutic value of FPs, particularly within traditional Chinese medicine. For instance, more than 1100 FPs prescriptions are documented in the Compendium of Materia Medica, and FPs are also widely used among ethnic minorities in China, such as fresh *Allium sativum* L. is used by the Tujia minority to treat foodborne stomach pain [1]. Similar practices are observed internationally. The *Ebers Papyrus* of Egypt documents the use of fresh *Aloe vera* L. for wound healing, while Ayurvedic texts highlight the application of fresh *Curcuma longa* L. in the treatment of inflammatory disorders. Together, these cross-cultural records underscore the longstanding recognition of FPs and provide a foundation for contemporary scientific research.

**Figure 1:**
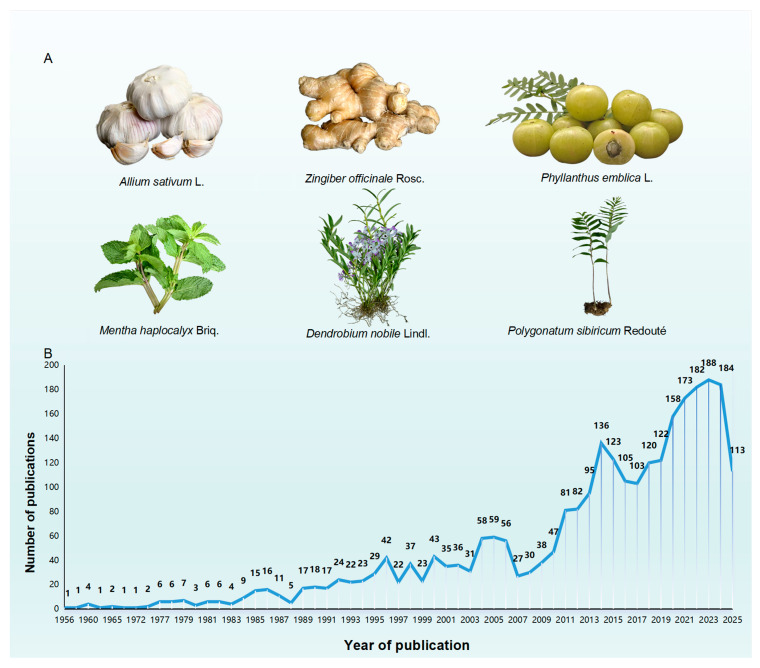
Representative plants in FPs (**A**). The number of publications between 1956 and 1 January 2026, at Web of Science database (http://apps.webofknowledge.com) and China National Knowledge Infrastructure (CNKI) by topic-based searching: “fresh herb” or “fresh” (**B**).

Despite their extensive historical use, the biomedical relevance of FPs has only recently attracted scientific attention. Recent studies demonstrate that FPs exhibit antimicrobial, antioxidant, hepatoprotective, and anticancer activities in standardized experimental models. Such findings support the effectiveness of investigating plant-based therapeutics within a contemporary biomedical framework. According to major databases such as PubMed, Web of Science, and the China National Knowledge Infrastructure (CNKI), over 800 FP-related publications have been published in the past five years (Figure 1). Accumulating experimental evidence indicates that dried phytomedicines (DPs) are not clinically interchangeable with FPs, as drying and processing can cause the degradation of labile constituents and the transformation or enrichment of specific bioactive compounds. Accordingly, FPs should not be viewed merely as unprocessed precursors of DPs but rather as a distinct pharmacological category with unique therapeutic potential, particularly for complex, multifactorial diseases. However, from a practical and logistical standpoint, DPs continue to dominate clinical practice because the preservation and transportation of FPs remain technically challenging. These practical constraints have also contributed to fragmented research efforts and a lack of synthesis from a modern biomedical perspective. As a result, systematically investigating the biomedical relevance and context-dependent advantages of FPs represent a timely and scientifically important endeavor, particularly given the current gaps in comparative and clinical evidence.

Although numerous studies have investigated individual FPs or specific biological activities, reviews focusing explicitly on FPs from a modern biomedical perspective remain limited. Existing reviews often address medicinal plants in general or focus predominantly on DPs, without critically comparing FPs and DPs under comparable experimental conditions. Moreover, critical aspects related to disease specificity, chemical composition differences, pharmacological mechanisms, dosage heterogeneity, safety considerations, and translational challenges have not been systematically integrated into a unified analytical framework. It remains unclear which disease contexts are most suitable for FP-based interventions and what mechanistic factors underlie their reported advantages over DPs. These gaps constrain a comprehensive understanding of FPs and hinder the development of evidence-based rationales for their clinical application.

Accordingly, this review aims to integrate historical and ethnomedicinal knowledge with contemporary biomedical evidence on the use of FPs. We summarize the global historical and traditional medical applications of FPs, followed by a bibliometric analysis to delineate research trends and knowledge structures in this field. Moreover, we also compare the chemical composition of FPs and DPs and compile evidence on their major pharmacological activities under comparable experimental conditions. Finally, we discuss safety concerns, methodological limitations, and emerging strategies, including advanced preservation technologies and green nanomaterial-based delivery systems, to provide a comparative, organized overview of current research on FPs in modern medicine.

## 2. Historical Overview

FPs have long served as cornerstones of traditional and folk medicine across diverse cultures. Classical medical records from major ancient civilizations reveal a systematic understanding of their preparation and therapeutic use (Table 1). Historical and ethnomedicinal evidence suggests that FPs have been valued for their rapid onset of action and perceived therapeutic effects. This longstanding, cross-cultural use provides historical context for contemporary pharmacological research, offering a basis for further scientific investigation.

### 2.1. Records in Medical Classics

The use of FPs has been widely documented across diverse regions and medical traditions. In China, the earliest recorded use of FPs dates back to the Prescriptions for Fifty-Two Diseases, which describes the application of fresh *Dioscorea oppositifolia* L. for the treatment of hemorrhoids. Subsequent classics, such as Shennong Bencao Jing, emphasized the use of FPs for heat-clearing and detoxification, laying an early theoretical foundation for their use. Concurrently, the Treatise on Febrile Diseases and Zhou Hou Bei Ji Fang further refined the clinical use of FPs. Qian Jin Yi Fang highlighted the efficacy of FPs in hemostasis and gynecological disorders during the Tang Dynasty. The Ming Dynasty marked the peak of FPs’ utilization. Compendium of Materia Medica documented more than 1000 prescriptions involving FPs. Moreover, physicians specializing in epidemic febrile diseases, such as Wang Shixiong, used fresh Houttuynia cordata Thunb. and other FPs in their treatments.

This systematic understanding of FPs was a global phenomenon. In ancient Egypt, the *Ebers Papyrus* (c. 1550 Before the Common Era) detailed fresh *Allium sativum* L., *Cannabis sativa* L., and *Aloe vera* (L.) Burm.f. for wound healing and infections. Similarly, the Ayurvedic texts Charaka Samhita and Sushruta Samhita documented over 1000 FPs [14]. Such historical evidence provides important context for understanding their longstanding use and supports further scientific investigation into their chemical and pharmacological characteristics.

### 2.2. Records in Traditional Ethnomedical Medicine

Ethnomedical records have long documented the therapeutic significance of FPs, especially in the ethnomedicinal systems of China’s ethnic minorities. The Dai minority treats mumps with freshly crushed leaves of *Polyscias sutchuenensis* and adds fresh *Houttuynia cordata* Thunb [15,16]. The Tujia minority uses fresh *Allium sativum* L. for foodborne stomach pain [1]. The Baise Zhuang minority employs a formulation comprising fresh *Andrographis paniculata*, *Houttuynia cordata*, and *Eriobotrya japonica* leaf juice for inflammatory conditions [17].

The pharmacological basis of these traditional practices has been investigated in modern studies. Fresh garlic has been shown to retain higher allicin content and exhibit potent antibacterial activity against foodborne pathogens, such as *Escherichia coli*, in vitro, suggesting a plausible mechanism for its traditional use in gastrointestinal in [1,18]. Similarly, the ethnomedical practice of the Baise Zhuang minority has been the subject of a preliminary clinical study, in which the formulation yielded a total effective rate of 97.50%, compared to 85.53% in the control group (*p* < 0.05) [17]. Furthermore, studies on Miao medicinal plants, such as *Balanophora subcupularis* P.C. Tam and *Sedum aizoon* L., have reported variations in the content of active constituents between FPs and their DPs [19,20,21,22].

It is important to distinguish between traditional uses documented in ethnomedical records and pharmacological activities validated by modern experimental approaches. The traditional practices described above reflect historical and cultural knowledge accumulated over centuries and provide valuable clues for hypothesis generation and guide the selection of plant materials for further pharmacological investigation. The following sections of this review examine the chemical composition and pharmacological properties of FPs using modern analytical and experimental methods, thereby providing a more rigorous basis for evaluating the therapeutic potential suggested by traditional knowledge.

## 3. Bibliometric Analysis

### 3.1. Literature Search Strategy

**Figure 2:**
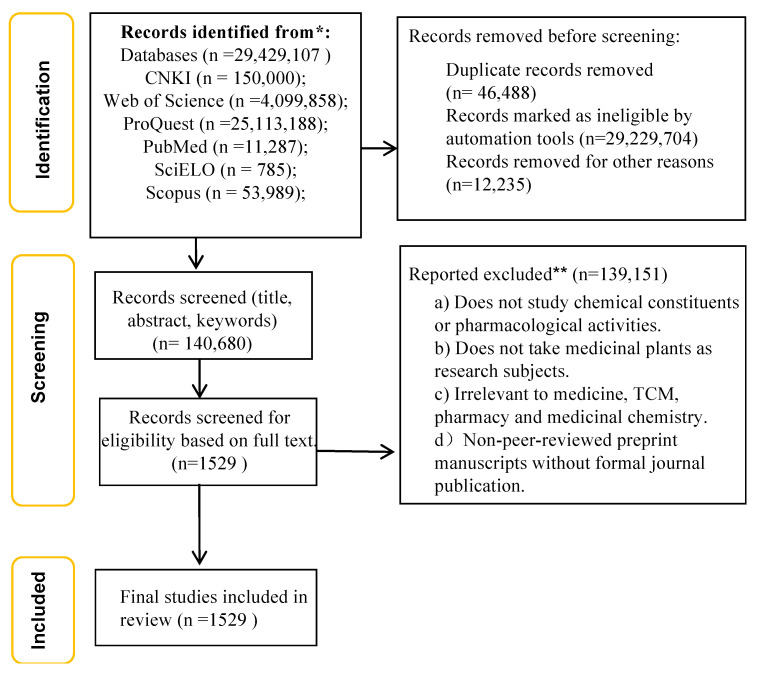
PRISMA diagram indicating the selection and filtering process. * the number of records identified from each database or register. ** indicate how many records were excluded.

We conducted systematic literature searches up to 3 July 2026 across PubMed, Web of Science, CNKI, ProQuest, SciELO and Scopus using “fresh” as the core keyword. Built-in disciplinary filters retained publications focusing on traditional Chinese medicine, pharmacology, pharmacy and medicinal chemistry, and all screening complied with PRISMA guidelines illustrated in Figure 2. In total, 29,429,107 initial records were collected, including 150,000 from CNKI, 4,099,858 from Web of Science, 25,113,188 from ProQuest, 11,287 from PubMed, 785 from SciELO and 53,989 from Scopus. A three-step pre-elimination was performed prior to manual screening: 46,488 duplicates were removed by reference management software, 29,229,704 irrelevant records were filtered automatically, and 12,235 non-qualifying items like conference abstracts, patents and papers without full texts were excluded, yielding 140,680 unique records for title and abstract assessment.

Citations were filtered by predefined criteria to discard research-lacking medicinal plant subjects, phytochemical or pharmacological analyses, off-discipline content, and unpublished preprints, which removed 139,151 records and reserved 1529 papers for full-text evaluation. Two researchers independently evaluated full-text quality, resolving disagreements through discussion with a senior reviewer. Studies with flawed experiments including absent controls, ambiguous sample sizes, incomplete statistics and insufficient extractable raw data were marked for exclusion. No papers were eliminated after full-text appraisal, and all 1529 eligible studies were included for subsequent data extraction and synthesis.

### 3.2. Analysis of Keyword Co-Occurrence and Temporal Trends

**Figure 3:**
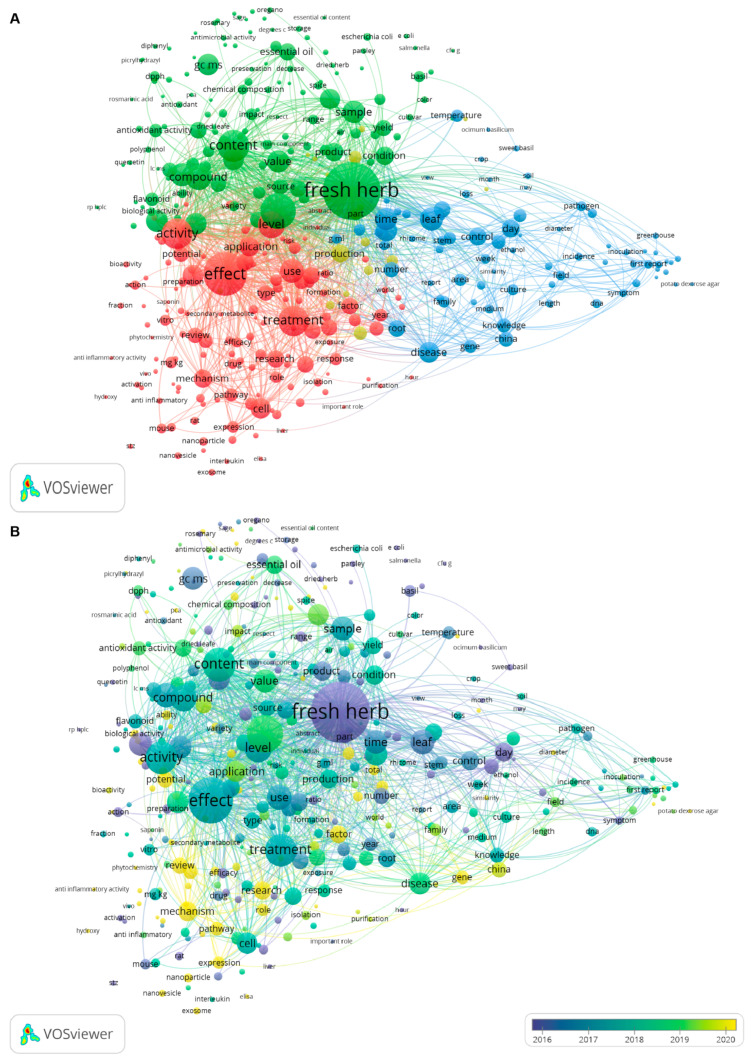
Keywords of co-occurrence analysis of FPs publications. Bibliometric network diagram derived from a literature search incorporating “fresh herb” and “fresh” across Web of Science, CNKI, PubMed, Scopus, ProQuest, and SciELO between 1956 and 1 January 2026. Different colors denote the four distinct clusters, each primarily characterized by high-frequency, high-impact terms: “chemical composition of FPs” (in green), “pharmacological activities and mechanisms” (in red), “disease-related therapeutic relevance” (in blue), and “methodological support for FP research” (in yellow) (**A**). Temporal keyword analysis of FPs publications, where node colors correspond to average publication year (purple: 2016, yellow: 2019) (**B**).

Visualization and network analyses were performed using VOSviewer, in which text-mining functions were applied to titles, abstracts, authors, citations, and keywords to extract relevant terms and generate co-occurrence maps. A total of 367 highly relevant keywords were selected for analysis. As illustrated in Figure 3, the size of each keyword node is proportional to its frequency of occurrence, whereas the thickness of the connecting lines represents the strength of co-occurrence between terms across the analyzed publications.

The keywords were clustered based on co-occurrence (Figure 3A). The prominent green cluster includes core terms such as “fresh herb”, “compound”, “content”, and “essential oil”. This cluster contains terms related to the chemical composition of FPs, including essential oils and polyphenols. The red cluster centers on terms such as “effect”, “activity”, “treatment”, and “mechanism”. The red cluster contains terms associated with pharmacological activities, such as antimicrobial and anti-inflammatory effects, and related mechanisms. The blue cluster comprises keywords such as “disease”, “pathogen”, “symptom”, and “gene”. The co-occurrence of “gene” with disease-related terms in this cluster reflects a statistical association within the literature, driven by studies that simultaneously mention molecular-level descriptors and pharmacological outcomes. The yellow cluster includes terms such as “sample”, “value”, “yield”, and “analysis”. The yellow cluster contains terms related to experimental design, quantitative assessment, and analytical techniques.

Keywords related to chemical composition, such as “compound” and “content”, were present throughout the 2016–2020 period. In the later years, terms associated with functional investigation, for example “effect” and “mechanism”, also appeared with higher frequency (Figure 3B). Additionally, researchers are paying more attention to the potential characteristics of FPs, such as the preservation of active compounds and their potential influence on bioavailability, which may provide a reference for future research on plant-based therapeutics. While there has been growing interest in the traditional applications, pharmacological effects, and chemical compositions of individual FPs, systematic comparisons of their chemical compositions and pharmacological effects with those of their DPs remain limited. Additionally, the safety profile of FPs remains underexplored.

## 4. Chemical Composition

**Figure 4:**
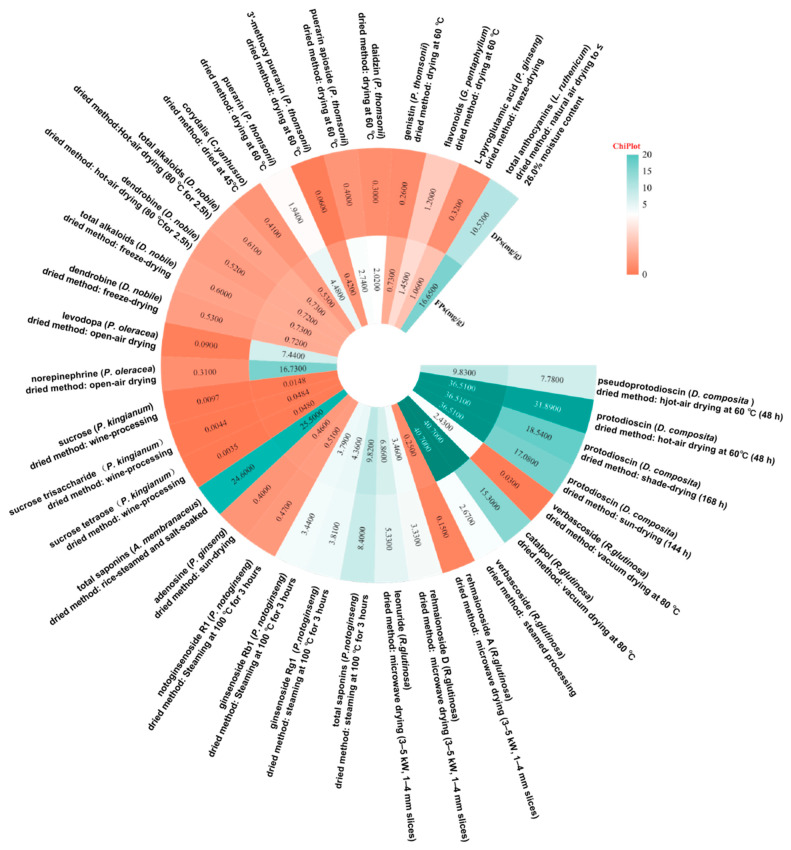
Chemical component concentration variability in FPs vs. DPs. The color scale represents the absolute concentration of each compound as reported in the original paired FPs/DPs.

The drying or processing of medicinal plants can significantly alter their chemical profiles, leading to either the loss, retention, or enrichment of specific compounds [23]. This section synthesizes studies comparing the chemical composition of FPs and DPs. The available evidence reveals a complex landscape: while certain compound classes, such as volatile oils and some polysaccharides, often show higher levels in FPs, other constituents may be enriched or newly formed during drying. Because the original publications use heterogeneous reporting units and do not consistently provide moisture content, extraction yield, or sample-weight basis, the quantitative values in Table 2 are presented as reported in the source studies. No cross-study normalization or conversion was performed. Therefore, Table 2 is intended to summarize paired fresh-dried comparisons within individual studies rather than to support direct quantitative comparisons across different studies. Where the reporting basis is not explicit in a source article, we do not infer it. This approach underscores the need for cautious, compound-specific interpretation of the data. Some bioactive compounds may be enriched in DPs due to chemical transformations during drying; therefore, the choice between FPs and DPs should be guided by the major active constituents and the specific therapeutic context, although such decisions currently remain constrained by limited comparative pharmacokinetic and clinical data [24]. In some cases, the observed differences between FPs and DPs are relatively minor, and for certain compounds, the direction of change is not consistent across species or drying methods (Figure 4).

### 4.1. Saponins

Saponins are among the key bioactive constituents of phytomedicines. Some studies indicate that FPs retain higher saponin content than DPs. Fresh *Panax ginseng* C. A. Meyer has a higher content of protopanaxadiol-type ginsenosides compared to *Panax ginseng* C. A. Mey dried at 80 °C. This difference may be attributed to the drying process breaking glycosidic bonds in ginsenosides [62,63]. Similarly, fresh *Panax notoginseng* (Burk.) F. H. Chen has a higher total saponin content compared to dried *Panax notoginseng* (Burk.) F. H. Chen. When fresh *Panax notoginseng* (Burk.) F. H. Chen is dried at 50 °C, the levels of saponin R1, ginsenoside Rg1, and ginsenoside Rb3 decrease by 1.00%, 12.60%, and 15.90%, respectively. Thermal drying progressively induces decarboxylation of malonyl saponins and hydrolytic cleavage of glycosidic bonds, leading to structural degradation [64]. Additionally, fresh *Dioscorea collettii* Hook.f. (*D. collettii*) has higher dioscin content than *D. collettii* sun-dried for 144 h. Specifically, the dioscin content in fresh *D. collettii* and dried *D. collettii* is 36.50 mg/g and 17.08 mg/g, respectively [25]. Furthermore, fresh *Radix Astragali* roots exhibited significantly higher concentrations of total saponins compared to oven-dried *Radix Astragali* dried at 100 °C. Specifically, the content of total saponins in fresh *Radix Astragali* and dried *Radix Astragali* is 25.50 mg/g and 24.60 mg/g, respectively [46]. Collectively, these findings indicate that certain saponin-rich FPs preserve higher levels of saponins compared with their dried counterparts, a difference that may have implications for the preparation of saponin-enriched extracts. Given their higher saponin retention, some FPs may serve as advantageous starting materials for obtaining saponin-rich fractions, although the in vivo significance of these compositional differences remains to be established. It should be noted, however, that the examples discussed above represent cases in which saponins are better preserved in fresh materials. This selection does not imply that all saponin-containing FPs consistently outperform their DPs.

### 4.2. Polysaccharide

Polysaccharides are the bioactive constituents of some FPs, which demonstrate a superior capacity compared to DPs in preserving the content and structural integrity of polysaccharides. This advantage is evident in several representative FPs, including *Dendrobium officinale* Kimura & Migo, *Helianthus tuberosus* L., and *Rehmannia glutinosa* (Gaertn.) Libosch. [65]. For instance, the polysaccharide content is 512.70 mg/g in fresh *Dendrobium officinale* Kimura et Migo versus 348.70 mg/g in *Dendrobium officinale* Kimura et Migo dried at 65 °C, and the mannose content is 489.50 mg/g versus 291.30 mg/g, respectively. Furthermore, fresh *Dendrobium officinale* Kimura et Migo exhibits superior polysaccharide integrity, characterized by a dominant component of approximately 1572 kDa. In contrast, dried *Dendrobium officinale* Kimura et Migo shows degradation into smaller fragments, including those of 583 kDa and 8 kDa. The mannose ratio also shifts from 1:0.83 in fresh *Dendrobium officinale* Kimura et Migo to 0.72:1 in dried *Dendrobium officinale* Kimura et Migo. The high molecular weight, polysaccharide integrity, and specific mannose ratio are directly linked to the superior in vitro and in vivo antioxidant activities of fresh *Dendrobium officinale* Kimura et Migo compared to dried *Dendrobium officinale* Kimura et Migo [66]. Similarly, fresh *Helianthus tuberosus* L. tubers retained 367.70 mg/g of polysaccharide and 183.30 mg/g of reducing sugar versus only 66.20 mg/g of polysaccharide and 15.20 mg/g of reducing sugar in Lactobacillus-cured *Helianthus tuberosus* L. [67]. Additionally, the stachyose content in fresh *Rehmannia glutinosa* Libosch roots is significantly higher than that in processed *Rehmannia glutinosa* Libosch. This difference may be attributed to the hydrolysis of polysaccharides in fresh *Rehmannia glutinosa* Libosch into oligosaccharides and monosaccharides during the drying process, which alters its traditional medicinal properties [68]. Collectively, these findings indicate that certain FPs can better preserve the quantity and molecular architecture of polysaccharides compared with their dried or processed counterparts.

In the case of *Dendrobium officinale* Kimura et Migo, such preservation has been linked to enhanced antioxidant activities in vitro and in vivo. However, for many other polysaccharide-rich botanicals, direct evidence linking compositional preservation to biological activity remains limited. Further studies are therefore needed to determine whether these chemical differences translate into meaningful pharmacological advantages. However, the preservation advantage of FPs is not universally observed across all polysaccharide-rich herbs. In some cases, processing or drying can actually increase polysaccharide yields or modify their structural features in ways that may affect bioactivity. For instance, the yield of polysaccharides from *Rehmannia glutinosa* Libosch increased from 0.55% in FPs to 4.45% in steam-dried products and further to 7.14% in repeatedly steamed and DPs, a more-than-12-fold increase compared with FPs [69]. This enhancement is attributed to high-temperature steaming increasing cell permeability and facilitating polysaccharide extraction [66]. In *Astragalus membranaceus,* honey processing led to a reduction in molecular weight from 2047.76 Da to 1695.79 Da, along with an increased glucose content in the monosaccharide composition [70]. Furthermore, the uronic acid content of *Polygonatum sibiricum* polysaccharides showed a tendency to first increase and then decrease with extended steaming time, indicating that processing does not simply degrade polysaccharides but may also generate structural variants with different physicochemical properties [71]. These examples further demonstrate that the effects of drying and processing on polysaccharides are highly species- and method-dependent, and a generalized claim of superior polysaccharide preservation in FPs across all species is not supported by the available evidence.

### 4.3. Alkaloids

Alkaloids are the main active ingredients in many *phytomedicines*. Some FPs exhibit higher alkaloid content compared to DPs. For instance, fresh *Portulaca oleracea* L. contains significantly higher levels of norepinephrine than air-dried *Portulaca oleracea* L. The norepinephrine content is 16.73 mg/g in fresh *Portulaca oleracea* L. and 0.31 mg/g in dried *Portulaca oleracea* L. [36,72]. For example, fresh *Leonurus japonicus* Houtt. contains significantly higher levels of stachydrine and leonurine than *Leonurus japonicus* Houtt. dried at 50 °C by hot-air drying. Specifically, the stachydrine content is 19.86 mg/g in fresh *Leonurus japonicus* Houtt. and 13.51 mg/g in dried *Leonurus japonicus* Houtt., while the leonurine content is 1.04 mg/g in fresh *Leonurus japonicus* Houtt. and 0.89 mg/g in dried *Leonurus japonicus* Houtt. [73]. Additionally, the dendrobine and total alkaloid contents in fresh *Dendrobium nobile* Lindl. are significantly higher than those in *Dendrobium nobile* Lindl. dried at 80 °C. Specifically, the dendrobine content is 0.72 mg/g in fresh *Dendrobium nobile* Lindl. and 0.52 mg/g in dried *Dendrobium nobile* Lindl., and the total alkaloid content is 0.73 mg/g in fresh *Dendrobium nobile* Lindl. and 0.61 mg/g in dried *Dendrobium nobile* Lindl. These changes may be attributed to thermal drying, which induces oxidative dehydrogenation and results in irreversible degradation of alkaloids [39]. Conversely, some DPs can contain higher levels of certain alkaloids than their FPs. For instance, dried *Corydalis yanhusuo* contains significantly higher levels of protopine, coptisine, palmatine, berberine, and dehydrocorydaline than fresh *Corydalis yanhusuo* from the same origin. In contrast, tetrahydropalmatine, a major bioactive alkaloid, is more abundant in FPs, suggesting that drying affects individual alkaloids in different ways [40]. In contrast, tetrahydropalmatine, a major bioactive alkaloid, is more abundant in the FPs, suggesting that drying affects individual alkaloids in different ways. Overall, these findings indicate that the effect of drying on alkaloid content is compound-dependent and species-dependent. While some alkaloids are better preserved in FPs, others may become enriched or even increase in content during the drying process. Therefore, the superiority of FPs over dried products cannot be generalized, and further research is needed to systematically investigate the stability, transformation, and net accumulation of individual alkaloids under different drying conditions.

### 4.4. Flavonoids

Some FPs, including fresh *Portulaca oleracea* L., *Plantago asiatica* L., and *Gynostemma pentaphyllum* (Thunb.) Makino exhibit higher flavonoid levels than their corresponding DPs. For example, the total flavonoid content in fresh *Portulaca oleracea* L. is 1.4-fold higher than in *Portulaca oleracea* L. dried at 80 °C. Specifically, the levels are 15.55 mg/g in fresh *Portulaca oleracea* L. compared with 10.75 mg/g in dried *Portulaca oleracea* L. Fresh *Portulaca oleracea* L. retains more flavonoid integrity because thermal drying promotes oxidative coupling and glycoside hydrolysis [74]. Similarly, fresh *Plantago asiatica* L. contains significantly higher total flavonoids than *Plantago asiatica* L. leaves dried at 60 °C. Fresh *Plantago asiatica* L. retains 167.80 mg/g of flavonoids, whereas dried *Plantago asiatica* L. contains 128.10 mg/g. This difference is likely due to thermal dehydration, which disrupts C-glycosidic linkages and oxidizes phenolic hydroxyls, leading to structural degradation [75]. The flavonoid content in fresh *Gynostemma pentaphyllum* (Thunb.) Makino leaves is significantly higher than in hot-air-dried *Gynostemma pentaphyllum* (Thunb.) Makino. The content of flavonoid is 1.18 mg/g in fresh *Gynostemma pentaphyllum* (Thunb.) Makino and 0.84 mg/g in dried *Gynostemma pentaphyllum* (Thunb.) Makino. This difference may be attributed to thermal drying, which induces oxidation, dehydrogenation, and isomerisation, thereby leading to irreversible degradation of bioactive flavonoids [39]. However, DPs can have higher flavonoid content than FPs in some plants and cases. For example, the rutin content in dried *Phyllanthus emblica* L. is approximately 4–5-fold higher than in fresh *Phyllanthus emblica* L., with levels ranging from 0.22% to 0.49% in dried samples compared with 0.05% to 0.10% in FPs across three producing regions [76]. Collectively, the effect of drying on flavonoids is compound- and species-dependent, and the superiority of FPs over DPs cannot be generalized. More robust studies are essential to validate this compositional complexity and determine its potential biological significance.

### 4.5. Volatile Oils

Volatile oils are one of the primary active constituents in aromatic FPs. FPs often exhibit higher volatile oil content than DPs, as thermal degradation during drying leads to significant losses of these heat-sensitive compounds. For example, the volatile oil content in fresh *Houttuynia cordata* Thunb. is significantly higher than in *Houttuynia cordata* Thunb. dried at 60 °C. Specifically, the volatile oil content is 12.50 mg/g in fresh *Houttuynia cordata* Thunb. and 5.00 mg/g in dried *Houttuynia cordata* Thunb. This difference may be attributed to the thermal degradation of unstable terpenes and esters during drying, leading to irreversible loss of aromatic compounds [77]. The volatile oil content of fresh *Zingiber officinale* Roscoe rhizomes is significantly higher than that of sun-dried *Zingiber officinale* Roscoe. Specifically, fresh *Zingiber officinale* Roscoe. contains 0.29 mg/g of volatile oil, compared to 0.22 mg/g in dried *Zingiber officinale* Roscoe. [48]. The content of cinnamaldehyde was 8.50 mg/g in fresh *Cinnamomum cassia* Presl bark and 8.04 mg/g in sun-dried *Cinnamomum cassia* Presl bark. Fresh *Cinnamomum cassia* Presl retained a higher cinnamaldehyde level because the drying process may cause thermal degradation of volatile compounds [78]. However, the impact of drying on volatile constituents is not uniformly deleterious across all compounds. A comparative GC-MS analysis of volatile components in fresh and dried Chuzhou *Chrysanthemum morifolium* Ramat. revealed that total volatile contents were 77.08% in FPs and 80.35% in DPs, indicating that drying does not necessarily reduce overall volatile detectability. For example, the n-hexadecanoic acid content in dried Chrysanthemum morifolium is significantly higher than in fresh Chrysanthemum morifolium, with levels of 8.63% in dried samples and 4.35% in FPs. These findings indicate that drying can both deplete and generate volatile constituents depending on the specific compound and drying conditions.

### 4.6. Other Compositions

Beyond the ingredients mentioned above, some FPs retain higher levels of other compounds compared to DPs. The *vitamin* C content of fresh *Citrus limon* (L.) Osbeck, *Origanum vulgare* L., and *Mentha haplocalyx* Briq. is more than 90% higher than that of DPs [60]. Compared to dried *Panax quinquefolius* L. (*P. quinquefolius*), fresh *P. quinquefolius* exhibits significantly higher levels of protein, vitamin C, vitamin E, volatile oil components, and superoxide dismutase activity, as determined by spectrophotometry and the pyrogallol autoxidation method (*p* < 0.01) (based on dry weight) [51]. The content of ferulic acid and caffeic acid in fresh *Portulaca oleracea* L. is higher than in dried *Portulaca oleracea* L. Specifically, ferulic acid content is 17.68 μg/g in fresh *Portulaca oleracea* L. compared with only 0.16 μg/g in dried *Portulaca oleracea* L., while caffeic acid content is 9.67 μg/g in fresh *Portulaca oleracea* L. and 0.13 μg/g in dried *Portulaca oleracea* L. [36]. The total tannin content in fresh *Phyllanthus emblica* L. is significantly higher than that in dried *Phyllanthus emblica* L. across different areas, including Fujian Zhangzhou, Yunnan Lincang, and Guangdong Chaoshan. Specifically, the tannin content in fresh *Phyllanthus emblica* L. ranges from 4.13% to 9.55%, while that in dried *Phyllanthus emblica* L. ranges from 2.99% to 8.69% [76]. The yields of water-soluble extracts and polyamide fractions were 3.00 g in fresh *Platycladus orientalis* (L.) Franco leaves and 2.50 g of dried leaves using the aqueous decoction–ethyl acetate extraction method. In contrast, this difference is crucial for understanding their pharmacological effects.

Overall, although some active constituents are present at higher levels in FPs than in DPs, it should be noted that the contents of active ingredients in FPs and DPs do not follow a uniform pattern in which FPs consistently exhibit higher levels. The effects of processing on plant chemistry are complex and can be broadly grouped into three patterns. One pattern is that some compound classes, such as polysaccharides and volatile oils, may show better total content preservation in either FPs or DPs, though individual constituents may deviate [76,78]. Another pattern is that many compounds show no significant differences between FPs and DPs. The third pattern is that a considerable number of compounds are enriched or even newly formed during drying. For instance, in *Dioscorea composita* Hemsl., HPLC-ELSD analysis of four saponins revealed that 60 °C hot-air drying caused the smallest content changes, whereas shade- and sun-drying led to a marked decrease in protodioscin but increases in diosgenin, pseudoprotodioscin, and polyphyllin I, illustrating that drying can both deplete and promote certain constituents [25]. Similarly, steaming *Polygonatum cyrtonema* transformed its polysaccharide from a linear fructan-dominated structure into a highly branched, galactan-rich form, demonstrating that processing can also induce structural modifications [79]. Overall, FPs and DPs are not chemically equivalent in a uniform manner; the direction and magnitude of changes are highly compound- and species-dependent, and even within the same herb, different compound classes may exhibit opposite trends. This variability arises from combined factors including thermal sensitivity, enzymatic activity, cellular disruption, and secondary metabolic conversions. Although such chemical divergences likely contribute to inconsistent pharmacological outcomes reported in preclinical studies, definitive causal links require rigorous comparative investigations. Given this complexity, comparisons between fresh and dried products should be assessed on a case-by-case basis, rather than asserting a universal superiority of one form over the other.

## 5. Biological Activities

**Figure 5:**
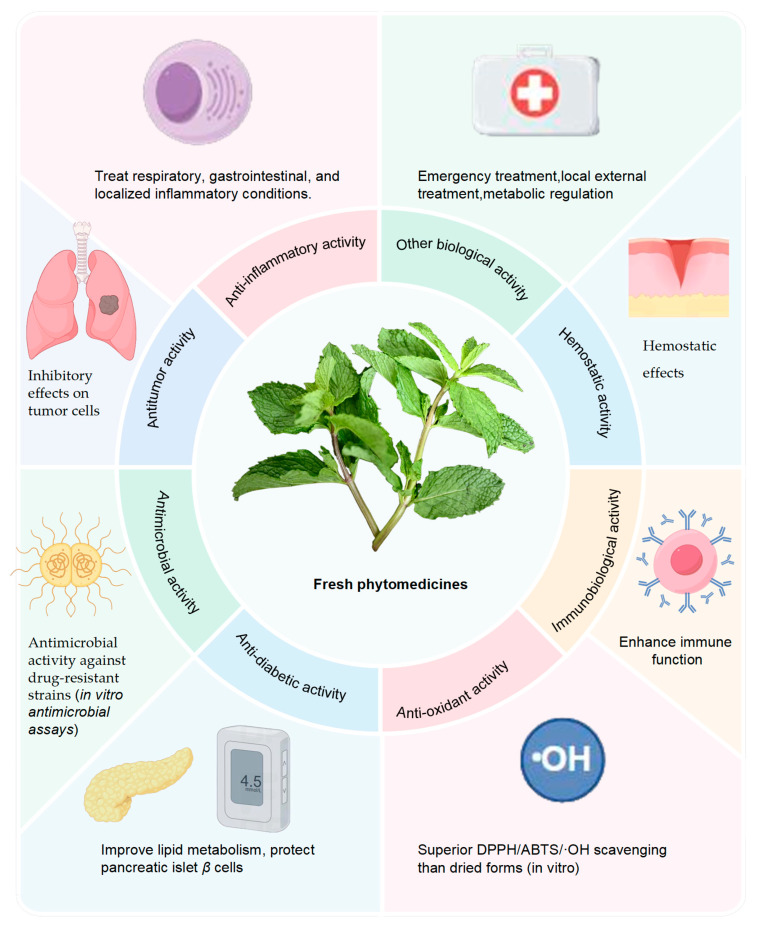
Main pharmacological activities of FPs. This schematic summarizes the major pharmacological activities of FPs reported in the literature, including antioxidant [30,49], anti-inflammatory [80,81], antimicrobial [18,78], antidiabetic [36,82,83], antitumor [84], hemostatic activity [85], and immunomodulatory effects [86,87,88] and other biological activities such as metabolic regulation. Most findings are derived from in vitro or animal studies and detailed experimental systems and animal models are summarized in Table 3.

FPs are increasingly recognized to possess distinct bioactive profiles and may provide novel directions for pharmacological research. Compared with DPs, certain FPs retain higher levels of bioactive components, which is reflected in their pharmacological efficacies in preclinical studies. Based on the collected evidence, the biological activities of FPs are most extensively reported in antimicrobial and antidiabetic effects, followed by antioxidant, immunoregulatory, antitumor, and anti-inflammatory activities. Notwithstanding these promising findings, the current evidence base is constrained by reliance on preclinical models, inter-study variability in experimental conditions, and a lack of clinical validation, warranting cautious interpretation and further standardized investigation. Collectively, available preclinical data indicate that FPs constitute a valuable research resource with distinct pharmacological characteristics and should not be regarded as functionally equivalent to DPs. A summary of reported biological activities is provided in Table 3 and Figure 5.

### 5.1. Antimicrobial Activity

Microbial infections remain a leading global health challenge, imposing significant economic burdens and public health concerns [89]. Numerous studies have reported that some FPs exhibit more potent antimicrobial activity than DPs under comparable experimental conditions. Although differences in chemical composition may contribute to these observations, the causal relationship between compositional changes and pharmacological outcomes has not been fully established. For example, at a concentration of 0.50 mg/mL, fresh *Portulaca oleracea* L. produced inhibition zones of 21.30 mm and 16.30 mm against *Shigella dysenteriae* and *Escherichia coli*, respectively, whereas dried *Portulaca oleracea* L. showed inhibition zones of 18.90 mm and 14.40 mm against the same two strains, respectively. This effect is likely due to the higher total flavonoid content in fresh *Portulaca oleracea* L. compared with dried *Portulaca oleracea* L., which can increase bacterial cell membrane permeability and decrease bacterial enzyme activity, thereby enhancing its antimicrobial efficacy [74]. Moreover, fresh *Allium sativum* L. showed more potent antimicrobial activity than pickled *Allium sativum* L. against a broader spectrum of *Staphylococcus aureus* (*S. aureus*), *E. coli*, *Bacillus subtilis* (*B. subtilis*), *Proteus vulgaris* (*P. vulgaris*), and *S. dysenteriae* [18]. Additionally, the essential oil of fresh and dried *Cinnamomum cassia* Presl showed minimum inhibitory concentrations of 0.27 ± 0.23 mg/mL and 0.46 ± 0.12 mg/mL against *S. aureus*, respectively. Minimum inhibitory concentrations were 0.27 ± 0.23 mg/mL and 0.35 ± 0.14 mg/mL against *E. coli*, respectively [78]. Collectively, these in vitro findings indicate the potential of certain FPs as promising sources of bioactive compounds for further antimicrobial research and development.

### 5.2. Antidiabetic Activity

**Figure 6:**
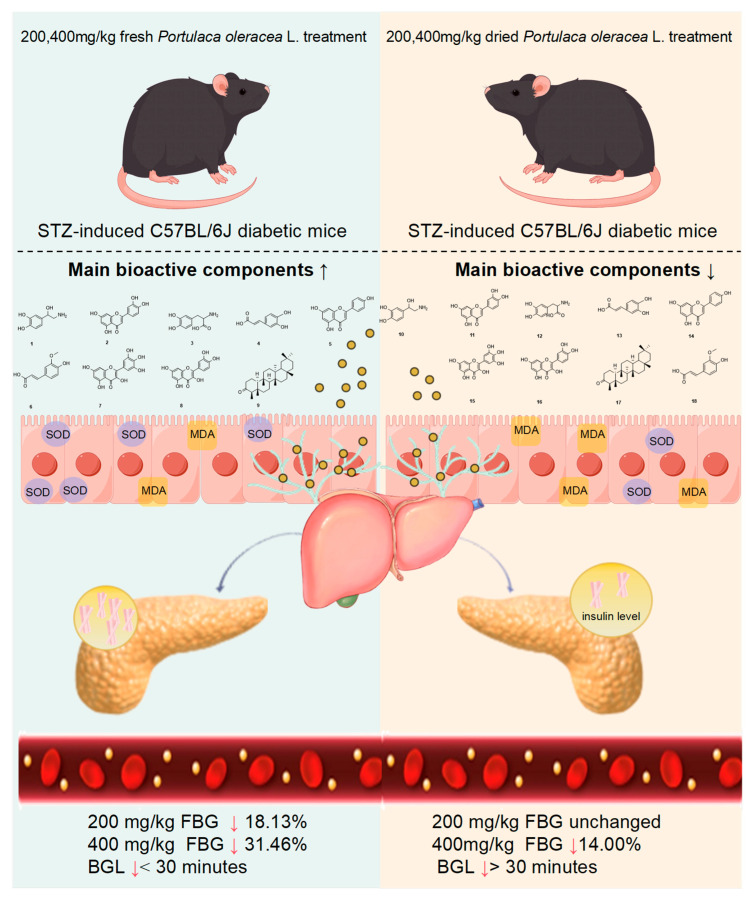
The main antidiabetic component and mechanisms of fresh *Portulaca oleracea* L. vs. dried *Portulaca oleracea* L. The figure was redrawn by the authors based on data from [36]. (BGL, blood glucose levels; FBG, fasting blood glucose; 1, norepinephrine; 2, luteolin; 3, levodopa; 4, caffeic acid; 5, apigenin; 6, ferulic acid; 7, myricetin; 8, quercetin; 9, fruedelin; 10, norepinephrine; 11, luteolin; 12, levodopa; 13, caffeic acid; 14, apigenin; 15, myricetin; 16, quercetin; 17, fruedelin; 18, ferulic acid). (↑: increase; ↓: decrease).

Diabetes is a common chronic endocrine disorder characterized by persistent hyperglycemia, and its incidence has gradually increased in recent years [90,91]. Accumulating evidence suggests that certain FPs may offer superior glycemic control efficacy compared with DPs in comparative animal studies. Although compositional differences between FPs and DPs have been proposed as a contributing factor, direct evidence linking specific chemical changes to the observed pharmacological advantages remains limited. This enhanced activity is corroborated by several preclinical studies that consistently link the superiority of FPs to their higher levels of specific active constituents. For instance, oral administration of extract from *Gynura divaricata* (L.) DC. at doses of 80 g/kg/day for 15 days, reduced fasting blood glucose in a high-fat diet/streptozotocin—induced type 2 diabetic mice, decreasing from 17.48 mmol/L to 8.85 mmol/L for fresh *Gynura divaricata* (L.) DC. and from 17.48 mmol/L to 12.17 mmol/L for dried *Gynura divaricata* (L.) DC. Fresh *Gynura divaricata* (L.) DC. also contained higher levels of polysaccharides than the DPs; however, direct evidence demonstrating that this compositional difference accounts for the observed antidiabetic advantage remains limited [82]. Furthermore, oral administration of an extract from *Portulaca oleracea* L. at 400 mg/kg/day for 3 weeks significantly reduced fasting blood glucose levels in Streptozotocin-induced C57BL/6J diabetic mice, with a 31.46% reduction in fresh *Portulaca oleracea* L. and 14.0% for dried *Portulaca oleracea* L., respectively. Additionally, fresh *Portulaca oleracea* L. significantly increased insulin levels compared to the dried *Portulaca oleracea* L. Fresh *Portulaca oleracea* L. contained higher concentrations of polyphenols and flavonoids than the DPs. These compositional differences may be associated with the observed pharmacological effects, although their causal contribution has not been fully established. Representative antidiabetic constituents and proposed mechanisms associated with fresh and dried *Portulaca oleracea* L. are summarized in Figure 6 [36]. Additionally, oral administration of fresh *Panax ginseng* C. A. Mey at 600 mg/kg/day for 8 weeks significantly reduced fasting blood glucose levels compared to dried *Panax ginseng* C. A. Mey in high-fat diet/streptozotocin-induced type 2 diabetic mice, likely due to the higher content of non-saponin components, such as adenosine and L-pyroglutamic acid, in fresh *Panax ginseng* C. A. Mey than in dried *Panax ginseng* C. A. Mey [83]. Overall, these studies indicate that FPs exhibit better antidiabetic activity in animal models, attributed to the preservation of critical active components that are often compromised during drying. This insight pinpoints specific active constituents for future research. Nevertheless, it is crucial to note that the current evidence is predominantly derived from animal models. Therefore, clinical trials are urgently required to validate the translational potential and clinical efficacy of FPs.

### 5.3. Antioxidant Activity

Oxidative stress refers to a state in which the balance between the body’s oxidative and antioxidant systems is disrupted. A growing body of in vitro evidence demonstrates that FPs exhibit superior antioxidant potency compared with DPs. Although differences in chemical composition may contribute to these observations, the mechanisms responsible for the reported activity differences have not been fully validated [92]. For instance, the extract of *Dendrobium officinale* Kimura et Migo at a concentration of 4.00 mg/mL exhibited significant 2,2-diphenyl-1-picrylhydrazyl (DPPH) radical scavenging activity and 2,2-azino-bis (3-ethylbenzothiazoline-6-sulfonic acid) radical cation (ABTS^+^) radical scavenging activity. The DPPH radical scavenging rates were 52.56% for fresh *Dendrobium officinale* Kimura et Migo and 41.34% for dried *Dendrobium officinale* Kimura et Migo. The ABTS^+^ radical scavenging rates were 61.24% for fresh *Dendrobium officinale* Kimura et Migo and 48.01% for dried *Dendrobium officinale* Kimura et Migo. These differences may be attributed to the higher polysaccharide and mannose contents in fresh *Dendrobium officinale* Kimura et Migo compared with dried *Dendrobium officinale* Kimura et Migo. Specifically, polysaccharide levels were 512.00 mg/g in fresh *Dendrobium officinale* Kimura et Migo and 489.50 mg/g in dried, while mannose levels were 348.70 mg/g and 291.30 mg/g, respectively [30]. Furthermore, the extracts of *Curcuma zedoaria* (Christm.) Roscoe at 10 mg/mL exhibited significant DPPH and ABTS^+^ radical scavenging activity. The DPPH radical scavenging rates were 83.96% for fresh *Curcuma zedoaria* (Christm.) Roscoe and 76.37% for dried *Curcuma zedoaria* (Christm.) Roscoe. The ABTS^+^ radical scavenging rates were 74.34% for fresh *Curcuma zedoaria* (Christm.) Roscoe and 62.33% for dried *Curcuma zedoaria* (Christm.) Roscoe. This may be due to higher levels of curdione and curcumol, at 21.33% and 11.73%, in fresh *Curcuma zedoaria* (Christm.) Roscoe, respectively, compared to 19.73% and 8.96% in dried *Curcuma zedoaria* (Christm.) Roscoe [49]. In addition, *Astragalus membranaceus* (Fisch.) Bge. (*Astragalus membranaceus* (Fisch.) Bge.) showed significant DPPH radical scavenging activity, ABTS^+^ radical scavenging activity, and hydroxyl radical (·OH) scavenging activity. At 10.00 mg/mL, DPPH radical scavenging rates were 77.18% for fresh *Astragalus membranaceus* (Fisch.) Bge. and 70.25% for dried *Astragalus membranaceus* (Fisch.) Bge.; at the same concentration, ·OH scavenging rates were 46.24% and 40.49%, respectively. At 20.00 mg/mL, ABTS^+^ radical scavenging rates were 77.18% for fresh *Astragalus membranaceus* (Fisch.) Bge. and 70.25% for dried *Astragalus membranaceus* (Fisch.) Bge. Fresh *Astragalus membranaceus* (Fisch.) Bge. had lower IC50 values than dried *Astragalus membranaceus* (Fisch.) Bge. across all three antioxidant assays. This difference is likely due to the higher content of active constituents in fresh *Astragalus membranaceus* (Fisch.) Bge. [93]. Overall, these studies suggest that FPs retain a more comprehensive profile of antioxidant compounds, leading to more robust in vitro activity. However, studies directly demonstrating their clinical antioxidant efficacy remain limited. Therefore, further research on FPs is essential to clarify their antioxidant potential.

**Table 3:** Summary of biological activities of FPs (↑: increase vs. control (*p* < 0.05); ↓: decrease vs. control (*p* < 0.05)). Evidence level key: In vitro = cell-based or biochemical assays. In vivo = animal models. Clinical = human studies.

Biological Activities	FPs	Medicinal Parts	Biological Ingredient	Evidence Level	Testing Subjects	Doses/Duration	Effects/Mechanisms	Ref.
Anti-inflammatory	*Perilla frutescens* (L.) Britton	Leaves	Rosmarinic acid, ferulic acid, luteolin	In vitro	LPS-Treated RAW 264.7 cells	Fresh25, 100 µg/mL	NO production by inhibiting the expression of Inducible Nitric Oxide Synthase and COX-2, TNF-*α*, IL-6, and IL-1*β* ↓ FPs (NO production by 47% at 100 µg/mL) > DPs (NO production by 12% at 100 µg/mL)	[94]
	*Taraxacum officinale* F.H.Wigg.	Whole plant	Total flavonoids	Clinical	Gastritis patients	8 weeks	IL-1*β*, IL-6 ↓	[95]
	*Plantago depressa* Willd.	Whole plant	Flavonoids, triterpenes, ursolic acid, total flavonoids	Clinical	Clinical cases	30–120 g/kg	Good therapeutic effect on nephritis	[96]
	*Mosla chinensis* (L.) Maxim.	Whole plant	Polysaccharides	In vivo	Rat paw edema model	50, 100, 200 mg/kg	Reduced paw edema	[85,94]
	*Mosla chinensis* (L.) Maxim.	Whole plant	Polysaccharides	In vitro	RAW264.7 macrophage cells	2, 5, 10 μg/mL	Inhibited IL-6, IL-12, NO; promoted IL-4, IL-10	[85]
	*Dendrobium huoshanense* Z.Z.Tang & S.J.Cheng	Stem	Alkaloids, amino acids and derivatives, biphenyls, carboxylic acids and derivatives, fatty acids and derivatives, flavonoid glycosides, flavonoids, phenols, phenylpropanoids, sugars, pterocarpans, terpenoids, etc.	In vivo	Chronic atrophic gastritis rat model	Fresh: 3.50, 7.00, 14.00 g/kg; dried: 0.70, 1.40, 2.80 g/kg	IL-6 (*p* < 0.05, *p* < 0.01), IL-1*β* (*p* < 0.05, *p* < 0.01), TNF-*α* (*p* < 0.05, *p* < 0.01) ↓.	[81]
Antimicrobial activity	*Portulaca oleracea* L.	Aboveground part	Total flavonoids, polysaccharides, total alkaloids, quercetin	In vitro	*E. coli, Shigella* spp., *Salmonella* spp.	0.125, 0.25 g/mL	*E. coli, Shigella* spp. ↓ (MIC = 0.125–0.5 mg/mL against *E. coli*, *S. aureus*, and *C. albicans*)	[74]
	*Curcuma zedoaria* (Christm.) Roscoe	Leaves	Essential oil	In vitro	*E. coli*, *Salmonella* spp., *S. aureus*	-	*E. coli* > *Salmonella* spp. > *S. aureus*	[49]
	*Allium sativum* L.	Garlic cloves	Allicin	In vitro	*Staphylococcus aureus*, *E. coli*, *B.subtilis*, *P. vulgaris*, *S. dysenteriae*, *P. aeruginosa*, *S. Typhi*, *C. albicans*.	-	FPs > vinegar-pickled garlic.	[18]
	*Thymus pulegioides* L.	Stems and leaves	Thymol, thymol methyl ether, dextromethorphan, and ortho-cymene	In vitro	*Alternaria* spp., *Penicillium italicum*, *Trichothecium roseum*	-	*Alternaria* spp., *Penicillium italicum*, *Trichothecium roseum* ↓ (FPs > DPs)	[97]
	*Amygdalus persica* var. *Persica* f. *duplex* Rehd.	Flowers	Volatile oil components, mainly including aldehydes (e.g., benzaldehyde), ketones, phytone	In vitro	Four bacterial strains: *S. Typhi, E. coli, B. subtilis, S. aureus*	-	The MIC values were 0.051 mg/mL for *E. coli*, 0.031 mg/mL for *S. aureus*, 0.031 mg/mL for *B. subtilis*, and 0.125 mg/mL for *S. typhi*	[98]
	*Isatis indigotica* Fort. (Woad)	Root	Alkaloids, phenolic compounds, polysaccharides	In vitro	*E. coli, S. aureus*, *Pseudomonas aeruginosa*	Dried root: 0.031–1.00 g/mL; fresh root: 0.062–2.00 g/mL	The MIC values of fresh root against *E. coli* and *S. aureus* are higher than those of the dried root, and the Minimum Bactericidal Concentration values of fresh root are twice those of the dried root. For *P. aeruginosa*, the MIC value of fresh root is twice that of the dried root, and the Minimum Bactericidal Concentration value of fresh root is twice that of the dried root.	[99]
	*Isatis indigotica Fort.* (Woad)	Leaves	Alkaloids, phenolic compounds, polysaccharides	In vitro	*E. coli, S. aureus*, *Pseudomonas aeruginosa*	Dried leaves: 0.031–1.00 g/mL; fresh leaves: 0.125–4.00 g/mL	Fresh leaves have stronger antibacterial activity than dried: their MICs against *E. coli* (2.0 g/mL), S. aureus (1.0 g/mL), and *P. aeruginosa* (1.0 g/mL) are all 4× those of the dried leaves (0.5 g/mL, 0.25 g/mL, 0.25 g/mL, respectively).	[99]
Antidiabetic activity	*Panax ginseng* C. A. Mey	Root	Total saponins	In vivo	Type 2 diabetes mellitus rat model	600.00 mg/kg	Regulate glucose and lipid metabolism, protect pancreatic *β*-cells, and modulate inflammatory responses; activate the AMPK signaling pathway and insulin secretion. ↑	[83]
	*Houttuyni cordata* Thunb.	Aerial parts	Flavonoids	In vivo	Streptozotocin-induced diabetic mouse model	0.10 g/mL	The fasting blood glucose ↓, the serum insulin concentration, and the inhibition rate on *α*-glucosidase at high concentration (FPs > DPs) ↑	[100]
	*Gynura diricata* (L.) DC.	Whole plant	Acidic polysaccharide	In vivo	Streptozotocin-induced diabetic mouse model	40.00, 80.00 g/kg (fresh); 5.00, 10.00 g/kg	The fasting blood glucose, total cholesterol, triglycerides, low-density lipoprotein cholesterol, non-esterified fatty acids, and catalase ↓	[82]
Antioxidant activity	*Dendrobium officinale* Kimura et Migo	Stem	Polysaccharide	In vitro	DPPH radical scavenging rates, ABTS^+^ radical scavenging rates, ·OH scavenging rates	0.50–2.50 mg/mL	Scavenging DPPH radical scavenging rates (52.56%), ABTS^+^ radical scavenging rates (61.24%), and ·OH scavenging rates (28.71%), >DPs (41.34%, 48.01%, and 15.84%)	[30]
	*Dendrobium officinale* Kimura et Migo	Stem	Polysaccharide	In vivo	CCl_4_ induced acute liver injury in mice model	FPs 9.77 g/kg/d, DPs 2.00 g/kg/d	Total antioxidant capacity, glutathione, catalase, superoxide dismutase levels in mouse liver tissue and serum (FPs > DPs) ↑, Malondialdehyde levels (FPs > DPs) ↓	[80]
	*Portulaca oleracea* L.	Whole plant	Polysaccharide	In vitro	·OH scavenging rates, cerium (IV) ion	1.196 g/L	Scavenging ·OH scavenging rates (FPs > DPs); rate of cerium (IV) ion (FPs > DPs) ↑	[101]
	*Dendrobium nobile* Lindl.	Stem	Polysaccharide	In vitro	DPPH radical scavenging rates, ABTS^+^ radical scavenging rates, and total antioxidant effect	-	Scavenging total antioxidant activity (FPs > DPs) (*p* < 0.05)	[39]
	*Achyranthus aspera* L.	Roots and rhizomes	Flavonoids	In vivo	Anti-lipid peroxidation	40.00 g/kg·d	Lipid peroxidation in the liver and kidneys (FPs > DPs) ↓	[102]
	*Curcuma zedoaria* (Christm.) Roscoe	Leaves	Essential oil	In vitro	DPPH radical scavenging rates, ABTS^+^ radical scavenging rates, Linoleic acid peroxidation inhibition assay, egg yolk peroxidation inhibition assay	2.00, 4.00, 6.00, 8.00, 10.00 mg/mL	Scavenging DPPH radical scavenging rates, ABTS^+^ radical scavenging rates, Linoleic acid peroxidation inhibition assay, egg yolk peroxidation inhibition assay ↑	[49]
	*Perilla frutescens* (L.) Britton	Leaves	Rosmarinic acid, ferulic acid, luteolin	In vitro	Human peripheral blood mononuclear cells	12.50, 100.00 µg/mL	Intracellular reactive oxygen species in a dose-dependent manner (FPs > DPs) ↓	[94]
	*Mosla chinensis* (L.) Maxim.	Whole plant	Polysaccharides	In vitro	DPPH radical scavenging rates, ABTS^+^ radical scavenging rates,	0.08–0.80 mg/mL	scavenged DPPH radical scavenging rates and ABTS^+^ radical scavenging rates,	[85]
Hemostasis	*Rehmannia glutinosa* Libosch	Roots	Iridoids	In vivo	Aspirin causes prolonged clotting time in mice	2.50, 5.00, 10.00 g/kg	The coagulation time ↓	[86]
	*Mosla chinensis* (L.) Maxim.	Whole plant	Polysaccharides	In vivo	Mouse tail-cutting hemostasis model	35.00, 70.00, 140.00 mg/kg	Bleeding time ↓	[85]
Neuroprotection, cardiovascular, and cerebrovascular protection.	*Panax ginseng* C. A. Mey	Roots	Ginsenosides	In vitro	The PC12 cell damage model was induced by corticosterone.	0.125, 0.25, 0.50, 1.00 mg/mL (identical concentrations for both FPs and DPs)	The protection of the cell damage induced ↑ (FPs > DPs)	[103]
	*Panax ginseng* C. A. Mey	Roots	Ginsenosides	In vitro	The PC12 cell damage model was induced by glutamic acid.	0.125, 0.25, 0.50, 1.00 mg/mL (identical concentrations for both fresh and dried samples)	The protection of the cell damage induced ↑ (FPs > DPs)	[103]
	*Achyranthes bidentata* Blume	Roots	Triterpenoid, saponins, ecdysterone	Clinical	140 hypertensive patients (clinical observation)	Once a day	Liver/kidney, improves micro-circulation ↑	[104]
Others	*Sedum sarmentosum* Bunge	Whole plant	Tannins, flavonoids (e.g., kaempferol 7-O-(6-O-propanediol)-glucoside)	In vitro	Phospholipase A2enzyme activity assay using Deinagkistrodon venom	1 mg fresh herb concentrate = 48 mg fresh *Sedum sarmentosum* Bunge; 1 mg dried herb water extract = 22 mg fresh *Sedum sarmentosum* Bunge; 1 mg dried herb ethanol extract = 25 mg *Sedum sarmentosum* Bunge	Inhibition of phospholipase A2 enzyme activity is superior to dry extracts	[105]
	*Sedum sarmentosum* Bunge	Whole plant	Tannins, flavonoids (e.g., kaempferol 7-O-(6-O-propanediol)-glucoside)	In vivo	Male Kunming mice (intraperitoneal injection of venom)	1 mg fresh herb concentrate = 48 mg fresh *Sedum sarmentosum* Bunge; 1 mg dried herb water extract = 22 mg fresh *Sedum sarmentosum* Bunge; 1 mg dried herb ethanol extract = 25 mg *Sedum sarmentosum* Bunge	Each group contained 6 mice. Normal saline group showed 16.7% survival. Only fresh herb concentrate significantly elevated survival to 33.3% (*p* < 0.05). Fresh juice and dried herb water extract retained 16.7% survival, while dried herb ethanol extract exhibited 0% survival.	[105]
	*Scutellaria barbata* D. Don	Whole Plant	Flavonoids	Clinical	Human patients with snakebite	Fresh herb applied as needed	Rapid pain relief, significant reduction in swelling	[106]
	*Dendrobium huoshanense* Z.Z.Tang & S.J.Cheng	Stem	Polysaccharides, flavonoids	In vitro	Acute gastritis model in 72 SD rats	The administration concentration of fresh samples was 5 times that of dried samples. 3.5, 7.0, 14.0 g/kg (fresh)/0.7, 1.4, 2.8 g/kg (dried)	Liver triglycerides, serum total cholesterol, and low-density lipoprotein cholesterol levels ↓; Serum high-density lipoprotein cholesterol levels ↑	[81]

Abbreviations: ABTS, 2,2′-azino-bis(3-ethylbenzothiazoline-6-sulfonic acid); DPPH, 2,2-diphenyl-1-picrylhydrazyl; FPs, fresh phytomedicines; IL-1*β,* interleukin-1*β*; IL-6, interleukin-6; LPS, lipopolysaccharide; MIC, minimum inhibitory concentration; NO, nitric oxide; SOD, superoxide dismutase; TNF-*α*, tumor necrosis factor-alpha; TGF-*β*, transforming growth factor-beta.

### 5.4. Immunoregulatory Activity

Immunomodulation refers to the regulation of immune function through bioactive compounds that enhance specific and non-specific immune responses [107]. Certain FPs are superior to DPs in immunoregulatory activity in experimental models, which has been observed in several comparative studies. However, the specific compositional factors responsible for these differences remain to be systematically investigated. For instance, administration of fresh *Rehmannia glutinosa* Libosch at 0.25, 0.50, and 1.00 g/kg for 10 days significantly increased the phagocytic capacity and phagocytic index of peritoneal macrophages in prednisolone acetate-induced immunosuppressed mice compared with dried *Rehmannia glutinosa* Libosch. Moreover, fresh *Rehmannia glutinosa* Libosch at 0.25, 0.50, and 1.00 g/kg for 10 days enhanced alkaline phosphatase expression in splenic lymphocytes of thyroxine-induced immunosuppressed mice relative to dried *Rehmannia glutinosa* Libosch [86]. Additionally, fresh *Phragmites australis* (Cav.) Trin. ex Steud. (*P. australis*) administered at a dose of 22.50 g/kg/day could upregulate lymphocyte transformation and phagocytic activity and enhance natural killer cell activity more effectively than dried *P. australis*. The better effect is attributed to the higher polysaccharide content in fresh *P. australis* [87]. Fresh *Bletilla striata* (Thunb.) Rchb.f., administered at doses of 100, 200, and 400 μg/mL, could upregulate interleukin-6 secretion in dendritic cells and enhance phagocytosis in RAW 264.7 cells, demonstrating superior effects compared with dried *Bletilla striata* Thunb. The better effect may be attributed to the fact that fresh *Bletilla striata* Thunb. has a richer monosaccharide composition compared to dried *Bletilla striata* Thunb. [32]. In summary, these examples underscore the immunomodulatory potential of certain FPs in preclinical models. Further investigation into their immunoregulatory activity and underlying mechanisms may inform the future development of fresh herb-based preparations, although clinical translation remains to be established.

### 5.5. Antitumor Activity

Tumors are abnormal tissue masses formed by the uncontrolled growth of cells [108,109]. They can invade nearby tissues and spread to distant sites, thereby posing serious health risks [110]. Some FPs exhibit more potent antitumor effects compared to DPs. For instance, the fresh *Typhonium giganteum* Engl. (*T. giganteum*) significantly reduced tumor mass and increased both the thymus and spleen indices in S180 tumor-bearing mice, compared with alum-processed *T. giganteum* [84]. The water extract of fresh *Hypericum perforatum* L. exhibits significant growth inhibition, with 50% values of 73%, 77%, and 58% in K562, U937, and LN229 leukemia cell lines, respectively, which are superior to those of dried *Hypericum perforatum* L. [111]. Moreover, the fresh extract of *Solanum nigrum* L. at concentrations of 5, 10, 30, 50, and 80 mg/mL significantly inhibited the proliferation of CNE-1 cells in a dose-dependent manner for 24, 48, and 72 h [41,112]. Despite some FPs showing more potent antitumor effects than DPs, clinical validation of FPs’ better antitumor activity requires further research.

### 5.6. Anti-Inflammatory Activity

Inflammation is a complex biological response of the body’s tissues to harmful stimuli [113]. Some pharmacological studies demonstrate that FPs exhibit superior anti-inflammatory efficacy to that of DPs in preclinical models, which is attributed to the preservation of thermolabile constituents, such as higher levels of organic acids and flavonoids in FPs. For instance, the extraction of fresh *Perilla frutescens* (L.) Britton. (*P. frutescens*) leaves at 100 µg/mL significantly reduced NO production compared to dried *P. frutescens* leaves in LPS-stimulated RAW 264.7 cells. This effect is likely attributable to the higher fumalic acid and luteolin contents in fresh *P. frutescens* leaves, which more effectively inhibit NF-κB signaling by reducing protein phosphorylation than dried *P. frutescens* leaves [94]. In another study, oral administration of fresh *Hedyotis diffusa* Willd. at 4 μg/mL significantly reduced the release of tumor necrosis factor-α and IL-6 compared with dried *Hedyotis diffusa* Willd. in the xylene-induced mice ear swelling model [114]. For instance, one small-scale clinical study reported that oral administration of fresh *Taraxacum mongolicum* Hand.-Mazz. at 13.14 mg/kg/day for 8 weeks significantly reduced IL-1*β* and IL-6 release in patients with gastritis, with apparently greater efficacy than dried *Taraxacum mongolicum* Hand.-Mazz. This effect may be attributed to the higher total flavonoid content in fresh *Taraxacum mongolicum* Hand.–Mazz. [95]. Collectively, these findings suggest that certain FPs may offer advantages over DPs in anti-inflammatory activity in experimental models, although further studies, particularly clinical trials, are needed to confirm these observations and elucidate their mechanisms.

### 5.7. Other Biological Activities

Some FPs exhibit significant advantages over DPs in various therapeutic areas, including hemostasis, antiemetics, neuroprotection, cardiovascular and cerebrovascular protection, liver protection, and emergency care. For example, *Aloe vera* (L.) Burm.f. demonstrated notable hemostatic efficacy in patients undergoing single platelet donation. Specifically, 98.57% of volunteers in the *Aloe vera* (L.) Burm.f. group achieved hemostasis within 5 min, compared with 57.14% in the control group treated with sterile medical adhesive tape compression. Moreover, the effective hemostasis rate was 100% in the *Aloe vera* (L.) Burm.f. group and 92.86% in the control group [115]. Additionally, some FPs show better antiemetic effects than DPs. For instance, oral administration of fresh *Zingiber officinale* Roscoe. at 1.00, 2.00, and 4.00 g/kg significantly prolonged the latency to vomiting and reduced its frequency compared with dried *Zingiber officinale* Roscoe. in pigeons induced with CuSO_4_. This effect is attributed to the higher volatile oil content in fresh *Zingiber officinale* Roscoe. The volatile oil content is 1.20% *w*/*w* in fresh *Zingiber officinale* Roscoe., whereas it is 0.80% *w*/*w* in dried *Zingiber officinale* Roscoe. [116]. Some FPs exhibit neuroprotective and cardiovascular health benefits. In particular, water extraction of fresh *Panax ginseng* C. A. Mey at doses of 0.25, 0.5, and 1.0 mg/mL significantly improved the viability of PC12 cells damaged by 200 μmol/L corticosterone and 150 μmol/L hydrogen peroxide compared with dry *Panax ginseng* C. A. Mey. This effect is likely attributable to the higher concentration of water-soluble proteins in fresh *Panax ginseng* C. A. Mey [103]. Moreover, certain FPs, including fresh *Taraxacum officinale* Weber ex F.H.Wigg. and *Dendrobium huoshanense* Z.Z.Tang & S.J.Cheng, exhibit more substantial hepatoprotective effects in the liver injury model [117]. Furthermore, some FPs show greater efficacy than DPs in emergency care. For example, oral administration of *Sedum sarmentosum* Bunge at 0.50 g/animal significantly reduced snakebite-induced mortality in mice. The survival rate reached 33.3% with fresh *Sedum sarmentosum* Bunge, compared with 16.7% for dried *Sedum sarmentosum* Bunge water extract and 0% for dried *Sedum sarmentosum* Bunge ethanol extract. This more substantial therapeutic effect is likely due to the higher levels of bioactive compounds, such as tannins and flavonoids, in fresh *Sedum sarmentosum* Bunge, which inhibit PLA2 enzyme activity and regulate Ca^2+^ concentration to protect the coagulation system [105]. Overall, these findings highlight the significant therapeutic potential of some FPs. To further explore the pharmaceutical potential of these plants, systematic investigation of the pharmacological activities of different FPs is warranted.

**Figure 7:**
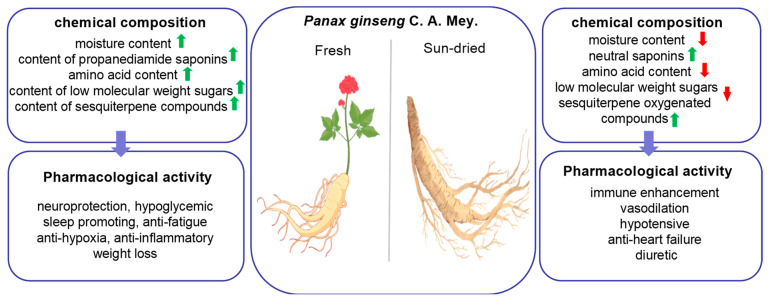
Analysis of reasons for differences in pharmacological activities between fresh *Panax ginseng* C. A. Mey. and sun-dried *Panax ginseng* C. A. Mey. (↑: increase; ↓: decrease).

Although FPs containing higher levels of thermosensitive bioactive compounds may exhibit superior pharmacological activities in certain contexts, such as acute conditions and antimicrobial, antioxidant, and antidiabetic settings, this advantage is not universal. The pharmacological activities of FPs are not inherently superior to those of DPs, but are determined by the specific active components involved and their transformation during drying. In certain cases, drying may enhance particular therapeutic effects by promoting the conversion or enrichment of active constituents. For example, dried *Panax ginseng* C. A. Mey has been reported to exhibit stronger anticancer and cardiovascular-protective activities than FPs, which may be associated with the conversion of malonyl-ginsenosides into more stable neutral ginsenosides, as well as the formation of unique volatile compounds during drying [118]. Figure 7 highlights the chemical composition and pharmacological activities of fresh *Panax ginseng* C. A. Mey compared with its dried *Panax ginseng* C. A. Mey. Similarly, dried *Dendrobium huoshanense* Z.Z.Tang & S.J.Cheng may show greater efficacy in chronic atrophic gastritis than the FPs, potentially due to enrichment in polysaccharides without structural degradation during drying in animal models [119]. Likewise, steamed *Polygonatum cyrtonema* Hua polysaccharides have been reported to exhibit superior antidiabetic activity compared with fresh polysaccharides, which is attributed to steam-induced structural remodeling that enhances gut microbiota fermentation and promotes the production of short-chain fatty acids, thereby improving glucose homeostasis [79]. Overall, whether FPs or DPs demonstrate superior efficacy is likely determined by the preservation, transformation, or enrichment of key bioactive components, as well as the pathological characteristics of the target disease.

## 6. Safety Concerns

As FPs gain popularity as conventional drugs and health products, their safety has become a critical concern. Available studies generally suggest that certain FPs exhibit low toxicity under experimental conditions. For instance, fresh *P. ginseng* showed no significant acute, genetic, or long-term toxicity in animal models, while *D. officinale* and *Phyllanthus emblica* L. also demonstrated low toxicity across tested dose ranges in preclinical studies [120,121,122].

However, it should be emphasized that such favorable safety profiles are not universal across all FPs. A number of FPs possess substantial inherent toxicity, and their cytotoxic and organotoxic effects may pose serious safety risks in clinical practice. A typical representative is *Pinellia ternata* (Thunb.) Makino, which is extensively recorded as unprocessed raw materia medica in classical works including Treatise on Cold-induced Diseases [123]. The fresh tuber of *Pinellia ternata* (Thunb.) Makino completely retains insoluble calcium oxalate raphides and irritant volatile alkaloids without degradation during storage or drying, which produce strong cytotoxicity against human oral, esophageal and gastric epithelial cells. Oral administration or external contact with unprocessed fresh pinellia readily induces severe adverse reactions such as mucosal erosion, throat swelling, persistent nausea and violent vomiting. By contrast, air drying, ginger processing and other processing methods can destroy these toxic crystals and decompose irritant alkaloids, thereby markedly reducing the acute toxicity and clinical risks of the corresponding DPs [124].

Consistent toxic variation rules can also be found in multiple toxic Araceae medicinal species suitable for FP application, including *Typhonium giganteum* Engl. and *Arisaema heterophyllum* Blume. Fresh tubers of the two herbs contain high levels of toxic calcium oxalate crystals and toxic terpenoid components; in vitro cell experiments confirm their stronger growth-inhibitory cytotoxicity to normal epithelial cells compared with processed DPs, and animal oral toxicity tests present evident gastrointestinal irritation and visceral injury symptoms [38,125].

Collectively, these comparative chemical and toxicological findings point to a fundamental limitation of FPs: the preservation of heat-labile components in FPs is inherently bidirectional. In retaining beneficial bioactive substances, FPs inevitably retain thermolabile toxic metabolites, which would normally be degraded or eliminated when drying is applied to produce DPs. This dual nature inevitably increases the risk of adverse reactions and clinical toxicity associated with unprocessed FPs.

What complicates this issue further is the scarcity of methodologically sound evidence to support a meaningful safety assessment of FPs. Most available toxicity studies have been conducted within narrow dose ranges and predominantly in animal models, which constrains the reliability of dose–response assessment and clinical extrapolation. Moreover, direct comparative safety studies between FPs and DPs are scarce. Existing investigations have largely focused on qualitative toxicity grading or mechanistic explanations of processing-induced detoxification, rather than providing quantitative or semi-quantitative comparisons of toxicity differences between fresh and dried forms. In addition, the high moisture content and lack of preservation processing in FPs may increase the risk of microbial contamination and chemical instability, while variations in plant origin, harvest time, and storage conditions further complicate their safety profiling.

Addressing these evidence gaps will require systematic safety evaluations that include direct comparisons with DPs, dose–response analyses, and long-term toxicity assessments.

## 7. Comparative Evidence, Limitations, and Strategic Perspectives

Based on a systematic integration and critical analysis of the available literature, this study identifies the advantages of FPs over DPs as reported in comparative preclinical studies. These advantages are observed under rigorously controlled, homogenized experimental conditions, including the use of the same botanical source, identical extraction procedures, equivalent dosing regimens, and comparable disease models across several core biomedical application domains [126]. In most cited studies, biological activities were investigated using relatively standardized in vitro and in vivo disease-relevant models, with antimicrobial effects commonly assessed by inhibition zone assays and minimum inhibitory concentration tests against Gram-positive and Gram-negative bacteria, antidiabetic activity evaluated in high-fat diet/streptozotocin-induced type 2 diabetic mouse models using fasting blood glucose, insulin, and lipid profiles as core endpoints, antioxidant capacity measured by DPPH, ABTS^+^, and hydroxyl radical scavenging assays, and anti-inflammatory effects tested in LPS-stimulated RAW 264.7 macrophages or xylene-induced ear edema models [23]. These advantages primarily arise from the fact that the chemical constituents of FPs often undergo irreversible loss or transformation during drying and processing. Consequently, FPs retain, to the greatest extent possible, thermolabile, volatile, and enzyme-sensitive bioactive components, including intact volatile oils, undegraded polyphenols (such as specific flavonoids and phenolic acids), endogenous active enzymes, and polysaccharides with preserved conformational structures [127]. Importantly, although higher levels of bioactive constituents are frequently associated with enhanced pharmacological activities, current evidence does not fully establish that compositional differences alone explain the reported advantages of FPs over DPs. Pharmacological outcomes may also be influenced by complex interactions among multiple constituents, their bioavailability, and other biological factors that remain incompletely understood. Collectively, these characteristics may contribute to the distinct biological properties observed in some FPs under specific experimental conditions. However, the extent to which individual compositional differences directly account for pharmacological advantages remains insufficiently validated, and additional mechanistic studies are required to establish causal relationships.

Beyond conventional chemical composition, plant-derived exosome-like nanovesicles (PELNs) have been successfully isolated from FPs using differential and ultracentrifugation methods, including *Platycodon grandiflorum* [128], purslane *Portulaca oleracea*, and *Andrographis paniculata* [129]. FPs serve as an ideal source for PELNs isolation, as they provide intact cells and native bioactive constituents [130]. Isolated PELNs from fresh *Platycodon grandiflorum* demonstrated significant anti-inflammatory and tissue-repair effects in an acute lung injury model through regulation of macrophage polarization [128], while fresh purslane-derived nanovesicles exhibited superior antioxidative, anti-inflammatory, and pro-proliferative properties, promoting diabetic wound healing in vivo [131]. These findings highlight that fresh plant-derived PELNs represent a promising source of bioactive nanocarriers with therapeutic potential. Importantly, they also suggest that the distinct biological activities of FPs may not be explained solely by differences in conventional phytochemical composition, providing a complementary mechanistic perspective for understanding the reported advantages of certain FPs.

**Figure 8:**
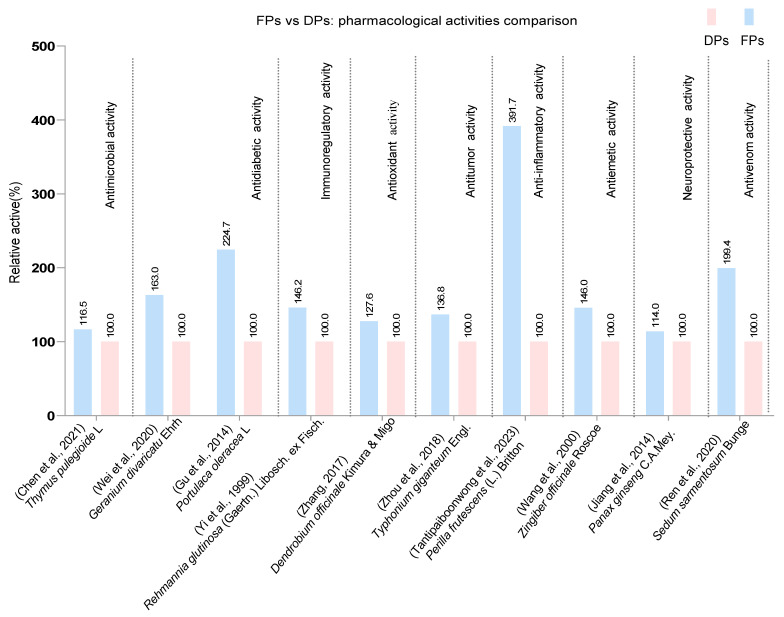
Fresh phytomedicines vs. dried phytomedicines: pharmacological activities comparison. Data are expressed as relative activity (%) normalized to dried phytomedicines (DPs = 100%), calculated by the formula: Relative activity (%) = (FPs activity index/DPs activity index) × 100%. Only paired homogeneous comparative studies meeting unified inclusion criteria were selected for plotting: fresh/dried phytomedicines share identical botanical origin, extraction protocols, dosage regimens and in vitro/in vivo experimental models within a single original study, with simultaneous quantifiable activity data for both FPs and DPs reported [30,36,82,84,86,94,97,103,105,116].

To provide a coherent and intuitive overview of representative findings reported in the literature, comparative outcomes derived from these standardized experimental paradigms are summarized in Figure 8. In contrast, the corresponding representative studies, experimental models, and evaluation endpoints are detailed in Supplementary Table S1. Overall, the use of relatively homogeneous, disease-relevant experimental frameworks enhances the comparability, reproducibility, and biomedical reliability of reported advantages of FPs over DPs. Nevertheless, the currently available evidence is still derived from a limited number of experimental systems and disease models, and therefore should not be generalized to all FPs or therapeutic contexts without further validation. On this basis, “freshness” can be regarded as a standardizable and quantifiable key quality attribute, suggesting that ‘freshness’ may represent a standardizable and quantifiable quality attribute that warrants further investigation as a basis for developing phytomedicinal preparations. In theory, FPs may offer a rational and tradition-consistent approach in therapeutic scenarios that rely on unstable bioactive constituents, where dried medicines exhibit insufficient efficacy, or within preventive medicine contexts associated with the concept of medicine–food homology. However, these potential applications require systematic investigation and clinical validation before they can be considered for clinical use. With further advances in stabilization technologies, standardized dosing studies, and targeted clinical validation, the advantages of FPs may eventually inform the development of clinically applicable preparations, although substantial translational challenges remain.

Nevertheless, the very characteristics that confer advantages to FPs—namely, chemical complexity and limited stability—also define their intrinsic limitations and restrict their applicability in specific biomedical contexts. Importantly, FPs are not inherently superior to DPs, and higher levels of individual constituents do not necessarily translate into superior pharmacological efficacy. In disease models in which therapeutic activity is driven predominantly by relatively stable secondary metabolites, drying or optimized processing may concentrate the active constituents, improve chemical stability, and ultimately enhance pharmacological efficacy. For example, in certain aromatic or phenylpropanoid-rich herbs, drying can selectively enrich stable components while reducing degradation associated with excessive moisture. Recent comparative evidence further demonstrates that, under well-controlled processing conditions, DPs may outperform their fresh counterparts in terms of bioactive compound abundance or pharmacological potency. Notably, repeated steam-drying and processing of *Rehmannia glutinosa* Libosch polysaccharides have been shown to enhance anti-inflammatory activity [65,119]. Collectively, these observations underscore that the rational selection between FPs and DPs should be guided by a mechanistic understanding of bioactive profiles, disease targets, and intended clinical outcomes, rather than by a generalized or uncritical preference for freshness.

## 8. Conclusions and Prospect

This review systematically integrates traditional knowledge and modern scientific evidence to elucidate the characteristics and therapeutic potential of FPs. The analysis indicates that FPs and DPs exhibit distinct pharmacological profiles, FPs may exhibit enhanced activity in specific domains, including antimicrobial, antioxidant, antidiabetic, immunomodulatory, antitumor, and anti-inflammatory effects under certain conditions. Such differences are closely associated with dynamic changes in chemical composition during processing, in which FPs generally retain higher levels of thermosensitive bioactive components, whereas drying may lead to degradation, enrichment, or transformation of specific constituents, thereby altering or even enhancing activities in DPs. Consequently, the therapeutic suitability of FPs and DPs may need to be evaluated according to specific disease contexts rather than assuming equivalence. Overall, the current evidence suggests that FPs and DPs are not directly interchangeable due to their distinct chemical and pharmacological profiles, highlighting the importance of rational selection and further systematic investigation in future research.

However, translating the intrinsic advantages of FPs into safe, effective, and controllable modern clinical practices remains challenging. These challenges represent the principal bottlenecks hindering their broader adoption, and specific scientific and practical issues accompany each.

(i) Lack of standardization and quality control. The chemical composition of FPs is highly dynamic, and dedicated quality evaluation frameworks are largely absent. In particular, establishing comprehensive quality control indices that simultaneously capture multi-component characteristics and biological activity faces significant methodological and standardization challenges. Future efforts should focus on developing specialized quality assessment systems by integrating metabolomics and biofingerprinting technologies, identifying quality markers that link chemical features to bioactivity, and implementing dynamic monitoring standards across the entire value chain, from cultivation to distribution.
(ii) Instability and limited supply capacity. FPs are inherently unstable and prone to spoilage, leading to frequent changes in their medicinal properties and difficulty meeting current clinical demand. Their preservation requires advanced storage and logistics systems, while existing stabilization technologies are often costly or difficult to scale, resulting in fragile supply chains and limited economic feasibility. Future research should prioritize advanced stabilization and formulation strategies, such as encapsulating key bioactive components using green nanocarriers, in combination with auxiliary technologies, including modified-atmosphere packaging and bio-coatings, to enhance stability while reducing costs and enabling industrial translation.
(iii) Insufficient research on dosage and pharmaceutical formulations. Dosage descriptions for FPs are inconsistent, and their multi-component, degradable nature challenges conventional pharmacokinetic models, making it difficult to characterize in vivo behavior and dose–effect relationships accurately. Future studies should conduct systematic investigations into dosage and pharmaceutics, develop novel pharmacokinetic models suitable for complex fresh medicinal systems, and promote standardized dosage expressions based on “dry-weight equivalence,” thereby providing a scientific basis for precision clinical use.
(iv) High-level clinical evidence is lacking. Clinical studies on FPs remain limited in number and heterogeneous in methodological rigor. Moreover, formulation instability and difficulties in placebo design pose substantial barriers to conducting high-quality randomized controlled trials. Future efforts should advance evidence-based clinical translation through innovative designs, such as pragmatic clinical trials, while strengthening collaborations among academia, industry, and healthcare institutions to overcome practical constraints in clinical research.
(v) Unclear rationale for choosing between “fresh” and “dried” forms. Due to insufficient comparative studies on the mechanistic effects of different processing methods and the complexity of clinical contexts, universally applicable and precise decision guidelines are lacking. Future research should establish a “fresh–dried–processed” rational selection knowledge base by integrating systematic comparative studies with big-data analytics, thereby clarifying optimal material forms for specific disease targets and providing scenario-based decision support for both clinical practice and drug development.

Some FPs exhibit distinct pharmacological and compositional advantages over DPs, as suggested by the integrated literature evidence. These advantages are primarily attributed to the ability of FPs to retain thermosensitive, volatile, and enzyme-labile bioactive constituents that are often degraded, lost, or chemically altered during the drying and processing of DPs, although emerging evidence suggests that plant-derived exosome-like nanovesicles may represent an additional mechanistic layer contributing to FP bioactivity plant-derived exosome-like nanovesicle from FPs appear to preserve native bioactivity more effectively than those from dried counterparts, and have demonstrated anti-inflammatory, antioxidant, and tissue-regenerative effects in preclinical models. However, the future advancement of FPs requires not only continued investigation of their pharmacological advantages, but also greater emphasis on quality standardization, formulation optimization, systematic characterization of PELN profiles, along with greater emphasis on quality standardization, formulation optimization, pharmaceutical research, and clinical evaluation to support their evidence-based development and future translational potential. In particular, priority should be given to representative FPs with substantial traditional use records and increasing modern scientific evidence, especially those documented in the Compendium of Materia Medica. Overall, this review systematically summarizes the traditional applications, chemical characteristics, pharmacological activities, and current limitations of FPs, while highlighting key strategies for improving their quality control, stability, pharmaceutical development, and progress toward clinical translation.

## Figures and Tables

**Table 1:** Historical records of applications of fresh phytomedicines.

No.	Plant Species	Traditional Applications	Cited in Classical Literature (Period/Dynasty)	Ref.
1	*Rehmannia glutinosa* Libosch	Nourish yin and blood, clear heat, treat hemorrhage	Han (206 BCE–220 CE) (Shennong Bencao Jing, Treatise on Cold-induced Diseases, Essential Prescriptions from the Golden Cabinet), Eastern Jin (317–420 CE) (Handbook of Prescriptions for Emergencies), Tang (618–907 CE) (Thousand Gold Prescriptions), Song (960–1279 CE) (Tai Ping Sheng Hui Prescriptions, Zheng Lei Ben Cao, Shengji Zonglu, Effective Prescriptions for Women’s Diseases), Ming (1368–1644 CE) (Qixiao Liangfang), Qing (1644–1912 CE) (Medical Records for Clinical Guidance, Discussion on Damp-Heat, Yi Zhen Yi De, Shi Bing Lun, Detailed Analysis of Epidemic Warm Diseases), Modern (20th–21st century) (Chinese Materia Medica Dictionary, Chinese Pharmacopeia)	[2,3,4,5,6,7,8,9,10,11,12,13]
2	*Zingiber officinale* Roscoe.	Relieve colds, suppress nausea/vomiting, improve digestion, alleviate cough, detoxify, stop bleeding	Han (206 BCE–220 CE) (Shennong Bencao Jing, Treatise on Cold-induced Diseases), Eastern Jin (317–420 CE) (Handbook of Prescriptions for Emergencies), Tang (618–907 CE) (Thousand Gold Prescriptions), Song (960–1279 CE) (Zheng Lei Ben Cao, Shengji Zonglu, Recipes for Saving Lives), Yuan (1271–1368 CE) (Danxi’s Essential Methods), Ming (1368–1644 CE) (Compendium of Materia Medica, Qixiao Liangfang), Qing (1644–1912 CE) (Medical Records for Clinical Guidance, Detailed Analysis of Epidemic Warm Diseases, Shi Bing Lun), Modern (20th–21st century) (Chinese Materia Medica Dictionary)	[2,3,4,5,6,7,8,9,10,11,12,13]
3	*Panax ginseng* C. A. Meyer	Treat fatigue, physical weakness, spleen deficiency, wasting thirst (diabetes), relieve stress	Han (206 BCE–220 CE) (Treatise on Cold-induced Diseases), Tang (618–907 CE) (Thousand Gold Prescriptions), Qing (1644–1912 CE) (Detailed Analysis of Epidemic Warm Diseases), Modern (20th–21st century) (Chinese Materia Medica Dictionary, Chinese Pharmacopeia)	[2,3,4,5,6,7,8,9,10,11,12,13]
4	*Glycyrrhiza uralensis* Fisch.	Relieve cough, detoxify, treat gastrointestinal disorders, asthma and peptic ulcers	Warring States (475–221 BCE) (Prescriptions for Fifty-two Diseases), Eastern Jin (317–420 CE) (Handbook of Prescriptions for Emergencies), Tang (618–907 CE) (Thousand Gold Prescriptions, Thousand Gold Pieces Formulary), Song (960–1279 CE) (Zheng Lei Ben Cao, Prescriptions of the Bureau of Taiping People’s Welfare Pharmacy), Ming (1368–1644 CE) (Qixiao Liangfang), Qing (1644–1912 CE) (Detailed Analysis of Epidemic Warm Diseases), Modern (20th–21st century) (Chinese Pharmacopeia)	[2,3,4,5,6,7,8,9,10,11,12,13]
5	*Coptis chinensis* Franch.	Treat diarrhea, dysentery, gastrointestinal inflammation, detoxify	Eastern Jin (317–420 CE) (Handbook of Prescriptions for Emergencies), Tang (618–907 CE) (Thousand Gold Prescriptions), Song (960–1279 CE) (Zheng Lei Ben Cao, Prescriptions of the Bureau of Taiping People’s Welfare Pharmacy), Ming (1368–1644 CE) (Qixiao Liangfang), Qing (1644–1912 CE) (Detailed Analysis of Epidemic Warm Diseases, Shi Bing Lun), Modern (20th–21st century) (Chinese Pharmacopeia)	[2,3,4,5,6,7,8,9,10,11,12,13]
6	*Phragmites australis* (Cav.) Trin. ex Steud.	Eliminate cough and phlegm, clear lung heat/fever, quench thirst, promote diuresis	Eastern Jin (317–420 CE) (Handbook of Prescriptions for Emergencies), Tang (618–907 CE) (Thousand Gold Prescriptions), Qing (1644–1912 CE) (Detailed Analysis of Epidemic Warm Diseases, Shi Bing Lun), Minguo (1912–1949 CE) (Dingganren medical case, Xing Lijiang Clinical Records), Modern (20th–21st century) (Chinese Materia Medica, Chinese Pharmacopeia)	[2,3,4,5,6,7,8,9,10,11,12,13]
7	*Aloe vera* (L.) Burm.f.	Relieve constipation, heal burns and skin lesions	Eastern Jin (317–420 CE) (Handbook of Prescriptions for Emergencies), Song (960–1279 CE) (Shengji Zonglu), Ming (1368–1644 CE) (Qixiao Liangfang), Qing (1644–1912 CE) (Shi Bing Lun), Modern (20th–21st century) (Chinese Pharmacopeia)	[2,3,4,5,6,7,8,9,10,11,12,13]
8	*Curcuma zedoaria* (Christm.) Roscoe	Resolve blood stasis, relieve pain, detoxify	Song (960–1279 CE) (Shengji Zonglu), Ming (1368–1644 CE) (Qixiao Liangfang), Qing (1644–1912 CE) (Shi Bing Lun), Minguo (1912–1949 CE) (Dingganren medical case)	[2,3,4,5,6,7,8,9,10,11,12,13]
9	*Nelumbo nucifera* Gaertn.	Stop bleeding, relieve heatstroke, treat hematuria	Eastern Jin (317–420 CE) (Handbook of Prescriptions for Emergencies), Tang (618–907 CE) (Thousand Gold Prescriptions), Song (960–1279 CE) (Tai Ping Sheng Hui Prescriptions, Effective Prescriptions for Women’s Diseases, Shengji Zonglu), Ming (1368–1644 CE) (Compendium of Materia Medica), Qing (1644–1912 CE) (Medical Records for Clinical Guidance, Integrating Chinese and Western Medicine), Minguo (1912–1949 CE) (Dingganren medical case, Xing Lijiang Clinical Records)	[2,3,4,5,6,7,8,9,10,11,12,13]
10	*Scutellaria baicalensis* Georgi	Reduce fever and inflammation, treat hemorrhoids, hemoptysis and hematemesis	Warring States (475–221 BCE) (Prescriptions for Fifty-two Diseases), Han (206 BCE–220 CE) (Shennong Bencao Jing), Tang (618–907 CE) (Thousand Gold Prescriptions), Song (960–1279 CE) (Zheng Lei Ben Cao), Ming (1368–1644 CE) (Qixiao Liangfang), Qing (1644–1912 CE) (Detailed Analysis of Epidemic Warm Diseases, Shi Bing Lun)	[2,3,4,5,6,7,8,9,10,11,12,13]
11	*Allium sativum* L.	Clear summer-heat, treat bacterial infections, protect cardiovascular system	Eastern Jin (317–420 CE) (Handbook of Prescriptions for Emergencies), Qing (1644–1912 CE) (Shi Bing Lun), Ancient Egypt (c. 1550 BCE) (Ebers Papyrus)	[2,3,4,5,6,7,8,9,10,11,12,13]
12	*Curcuma longa* L.	Treat skin infections, wounds and inflammatory disorders	Ancient India (c. 2nd cent. BCE–2nd cent. CE) (Charaka Samhita, Sushruta Samhita)	[2,3,4,5,6,7,8,9,10,11,12,13]
13	*Allium macrostemon* Bunge	Alleviate chest pain, spasms and gangrene	Warring States (475–221 BCE) (Prescriptions for Fifty-two Diseases), Eastern Jin (317–420 CE) (Handbook of Prescriptions for Emergencies)	[2,3,4,5,6,7,8,9,10,11,12,13]
14	*Lycium barbarum* L.	Nourish yin, improve blurred vision, relieve dizziness	Tang (618–907 CE) (Thousand Gold Prescriptions), Song (960–1279 CE) (Zheng Lei Ben Cao, Shengji Zonglu), Ming (1368–1644 CE) (Compendium of Materia Medica), Qing (1644–1912 CE) (Detailed Analysis of Epidemic Warm Diseases), Minguo (1912–1949 CE) (Dingganren medical case)	[2,3,4,5,6,7,8,9,10,11,12,13]
15	*Astragalus membranaceus* (Fisch.) Bge.	Alleviate fatigue and immune deficiency	Tang (618–907 CE) (Thousand Gold Prescriptions), Qing (1644–1912 CE) (Integrating Chinese and Western Medicine)	[2,3,4,5,6,7,8,9,10,11,12,13]
16	*Portulaca oleracea* L.	Nourish yin, relieve dry cough and fever	Tang (618–907 CE) (Emergent Prescriptions Worth Thousands of Gold), Modern (20th–21st century) (Chinese Materia Medica)	[2,3,4,5,6,7,8,9,10,11,12,13]
17	*Dendrobium nobile* Lindl.	Nourish yin, relieve dry cough, abate fever	Qing (1644–1912 CE) (Shi Bing Lun), Minguo (1912–1949 CE) (Xing Lijiang Clinical Records), Modern (20th–21st century) (Chinese Materia Medica, Chinese Pharmacopeia)	[2,3,4,5,6,7,8,9,10,11,12,13]
18	*Plantago asiatica* L.	Relieve cough, sore throat, edema and promote diuresis	Eastern Jin (317–420 CE) (Handbook of Prescriptions for Emergencies), Minguo (1912–1949 CE) (Dingganren medical case, Xing Lijiang Clinical Records)	[2,3,4,5,6,7,8,9,10,11,12,13]
19	*Artemisia annua* L.	Treat malaria, fever, inflammation and bleeding	Qing (1644–1912 CE) (Medical Records for Clinical Guidance), Modern (20th–21st century) (Chinese Materia Medica Dictionary, Chinese Pharmacopeia)	[2,3,4,5,6,7,8,9,10,11,12,13]
20	*Cinnamomum cassia* Presl	Disperse cold, relieve rheumatism and edema	Tang (618–907 CE) (Thousand Gold Prescriptions), Minguo (1912–1949 CE) (Xing Lijiang Clinical Records), Modern (20th–21st century) (Chinese Materia Medica)	[2,3,4,5,6,7,8,9,10,11,12,13]
21	*Isatis indigotica* Fortune	Resolve mental confusion, treat stroke, convulsions, hemoptysis and heatstroke	Song (960–1279 CE) (Zheng Lei Ben Cao, Shengji Zonglu), Minguo (1912–1949 CE) (Dingganren medical case), Modern (20th–21st century) (Chinese Materia Medica)	[2,3,4,5,6,7,8,9,10,11,12,13]
22	*Acorus tatarinowii* Schott	Resolve mental confusion, treat stroke, convulsions, hemoptysis and heatstroke	Warring States (475–221 BCE) (Prescriptions for Fifty-two Diseases), Eastern Jin (317–420 CE) (Handbook of Prescriptions for Emergencies), Song (960–1279 CE) (Zheng Lei Ben Cao, Shengji Zonglu), Ming (1368–1644 CE) (Compendium of Materia Medica), Qing (1644–1912 CE) (Shi Bing Lun), Minguo (1912–1949 CE) (Dingganren medical case)	[2,3,4,5,6,7,8,9,10,11,12,13]
23	*Taraxacum mongolicum* Hand.-Mazz	Relieve sore throat, cough, mastitis and conjunctivitis	Eastern Jin (317–420 CE) (Handbook of Prescriptions for Emergencies), Modern (20th–21st century) (Chinese Materia Medica Dictionary)	[2,3,4,5,6,7,8,9,10,11,12,13]
24	*Pueraria lobata* Radix	Alleviate fever, headache and hematemesis	Tang (618–907 CE) (Thousand Gold Prescriptions), Qing (1644–1912 CE) (Song Feng’s Discourse on Epidemics)	[2,3,4,5,6,7,8,9,10,11,12,13]

**Table 2:** Chemical composition comparison between FPs and DPs. Values are presented using the original units and quantitative bases reported in the source studies. No cross-study normalization or conversion was performed because moisture content, extraction yield, sample-weight basis, and reporting units were not consistently available across the original publications. Therefore, the table is intended to summarize paired fresh-dried comparisons within the same study rather than direct quantitative comparisons across different studies.

Chemical Composition	Plant Species	Detection Part	Drying Method	Detection Method	Chemical Composition Changes (FPs vs. DPs)	Ref.
Saponins	*Dioscorea composita* Hemsl.	Root stem	Hot-air drying at 60 °C (48 h)	Ultra-Performance Liquid Chromatography coupled with Quadrupole Time-of-Flight Mass Spectrometry (HPLC-ELSD)	Protodioscin 36.51 mg/g vs. 31.89 mg/g, pseudoprotodioscin 9.83 mg/g vs. 7.78 mg/g;	[25]
	*Dioscorea composita* Hemsl.	Root stem	Shade-drying (168 h)	HPLC-ELSD	Protodioscin 36.51 mg/g vs. 18.54 mg/g	[25]
	*Dioscorea composita* Hemsl.	Root stem	Sun-drying (144 h)	HPLC-ELSD	Protodioscin 36.51 mg/g vs. 17.08 mg/g	[25]
	*Rehmannia glutinosa* Libosch	Root	Vacuum drying at 80 °C	HPLC-UV, GC-MS	Verbascoside 0.0296–0.1090% vs. 0.0147–0.0465%; catalpol 0.8350–2.1500% vs. 0.0213–0.8050%;	[26]
	*Rehmannia glutinosa* Libosch	Root	Microwave drying (3–5 kW, 1–4 mm slices)	HPLC	Rehmaionoside A 0.25 vs. 0.15 mg/g, rehmaionoside D 3.46 vs. 3.33 mg/g, leonuride 6.86 vs. 5.33 mg/g	[26]
	*Panax notoginseng* (Burk.) F. H. Chen	Root and rhizome	Steaming at 100 °C for 3 h	HPLC-ELSD	Total saponins 9.82 mg/g vs. 8.40 mg/g, ginsenoside Rg1 4.36 mg/g vs. 3.81 mg/g, ginsenoside Rb1 3.79 mg/g vs. 3.44 mg/g, notoginsenoside R1 0.51 mg/g vs. 0.47 mg/g	[27]
Polysaccharide	*Rehmannia glutinosa* Libosch	Root	Dried	HPLC-ELSD	Stachyose: fresh 57.90% vs. 38.10%	[28]
	*Rehmannia glutinosa* Libosch	Root	Steamed	HPLC-ELSD	Stachyose 57.90% vs. 6.39%	[28]
	*Camellia sinensis* (L.) O. Kuntze cv.‘Bixiangzao’	Leaves	Oolong tea processing (withering vs. shaking vs. kill–green vs. rolling vs. drying)	HPLC, SPME/GC-MS	Soluble sugars 7.22% vs. 6.84%	[29]
	*Dendrobium officinale* Kimura et Migo	Stems	Hot-air drying at 65 °C	HPLC, Phenol-ulfuric acid method	Total polysaccharide content 512.70 vs. 348.70 mg/g, mannose content 489.50 vs. 291.30 mg/g	[30]
	*Trichosanthes kirilowii* Maxim.	Root	105 °C (hot-air drying)	Anthrone-sulfuric acid method	Soluble polysaccharide content 4.36% vs. 1.14%	[31]
	*Bletilla striata* Thunb.	Rhizome	105 °C (hot-air drying)	HPLC-ELSD, Ultraviolet-visible spectroscopy, FT-IR, SEM, Dynamic Light Scattering	Total polysaccharides 90.40% vs. 81.42%, uronic acid content: fresh 6.11% vs. dry 5.83%	[32]
	*Polygonatum kingianum* Coll. et Hemsl.	Root	Nine-steaming and nine-sunning	Spectrophotometry and HPLC	Polysaccharides 198.39 ± 17.96 mg/g vs. 66.87 ± 25.12 mg/g	[33]
	*Polygonatum kingianum* Coll. et Hemsl.	Root	Wine-processing	Ultra-Performance Liquid Chromatography coupled with Quadrupole Time-of-Flight Mass Spectrometry	Sucrose Tetraose 0.048 mg/g vs. 0.0035 mg/g. Sucrose Trisaccharide 0.0484 mg/g vs. 0.0044 mg/g. Sucrose 0.0148 mg/g vs. 0.0097 mg/g	[34]
	*Dendrobium officinale* Kimura et Migo	Leaf	Air-dried	UPLC-PDA, phenol-sulfuric acid method	Mannose 31.59% vs. 30.84%, polysaccharides 73.19% vs. 71.02%	[35]
	*Polygonatum kingianum* Coll. et Hemsl.	Leaf	Air-dried	UPLC-PDA, Phenol-Sulfuric Acid Method	Mannose 17.52% vs. 15.04%, polysaccharides 87.28% vs. 70.12%	[35]
Alkaloids	*Portulaca oleracea* L.	Root	Open-air drying	ATR-FTIR, Ion Chromatography	Norepinephrine 16.73 μg/g vs. 0.31 μg/g. Levodopa 7.44 vs. 0.09 μg/g	[36]
	*Leonurus heterophyllus* Sweet	Whole plant	Fried yellow product	Spectrophotometry, TLC	Total alkaloids 1.383% vs. 1.327%	[37]
	*Leonurus heterophyllus* Sweet	Whole plant	Wine-processed product	Spectrophotometry, TLC	Total alkaloids 1.383% vs. 1.331%	[37]
	*Leonurus heterophyllus* Sweet	Whole plant	60 °C 10 min wine-baked product	Spectrophotometry, TLC	Total alkaloids 1.383% vs. 1.300% (10 min). Total Alkaloids 1.383% vs. 1.300% (15 min)	[37]
	*Typhonium giganteum* Engl.	Tuber	Air-dried	HPLC, TLC, Spectrophotometry	Total alkaloids 0.19% vs. 0.16%	[38]
	*Dendrobium nobile* Lindl.	Stem	Freeze-drying (−20 °C pre-freezing for 1.5 h)	UPLC-Q-Orbitrap HRMS, GC, Ultraviolet-visible spectroscopy	Dendrobine 0.72 mg/g vs. 0.53 mg/g, total alkaloids 0.73 mg/g vs. 0.60 mg/g	[39]
	*Dendrobium nobile* Lindl.	Stem	Hot-air drying (80 °C for 2.5 h)	UPLC-Q-Orbitrap HRMS, GC, Ultraviolet-visible spectroscopy	Dendrobine 0.72 mg/g vs. 0.52 mg/g, total alkaloids 0.73 mg/g vs. 0.61 mg/g	[39]
	*Corydalis yanhusuo* (Y.H.Chou & C.C.Hsu) W.T.Wang ex Z.Y.Su & C.Y.Wu	Tuber	Dried at 45 °C	HPLC	Corydalis 0.53 mg/g vs. 0.41 mg/g	[40]
	*Solanum photeinocarpum* Nakamura et S. Odashima	Fruits	Air-dried	HPLC	Solasodine: 2.67 mg/g vs. 1.34 mg/g. Solanidine: 3.40 mg/g vs. 1.70 mg/g	[41]
Flavonoids	*Sedum aizoon* L.	Stem	Hot-air drying (80 °C)	UV Spectrophotometry (256 nm)	Total flavonoids 17.81 mg/g vs. 17.51 mg/g (spring), total flavonoids 53.24 mg/g vs. 27.63 mg/g (summer), total flavonoids 22.20 mg/g vs. 18.50 mg/g (autumn)	[19]
	*Sedum aizoon* L.	Root	Hot-air drying (80 °C)	UV Spectrophotometry (256 nm)	Total flavonoids 64.61 mg/g vs. 36.39 mg/g (spring), total flavonoids 61.82 vs. 36.57 mg/g (summer), total flavonoids 37.40 mg/g vs. 30.39 mg/g (autumn)	[19]
	*Taraxacum mongolicum* Hand.-Mazz.	Whole plant	Stir-frying to carbon	UV Spectrophotometry (508 nm)	Total flavonoids 3.90% vs. 2.90%	[42]
	*Pueraria thomsonii* Benth.	Root	Drying at 60 °C	HPLC (250 nm)	Puerarin 4.48 mg/g vs. 1.94 mg/g, 3′-methoxy puerarin 0.42 mg/g vs. 0.06 mg/g, puerarin apioside 2.74 mg/g vs. 0.40 mg/g, daidzin 2.02 mg/g vs. 0.30 mg/g, genistin 0.73 mg/g vs. 0.26 mg/g	[43]
	*Gynostemma pentaphyllum* (Thunb.) Makino	Aerial parts (stems and leaves	Dried at room temperature	Ultraviolet-visible spectroscopy Spectrophotometry	Flavonoids 1.45 mg/g vs. 1.20 mg/g	[44]
	*Lycium ruthenicum* Murr.	Fruit	Natural air drying to ≤26.0% moisture content	HPLC	Flavonols 1.21 μg/g vs. 0.74 μg/g, total anthocyanins 16.65 mg/g vs. 10.53 mg/g	[45]
	*Astragalus membranaceus* (Fisch.) Bunge	Root	Rice-steamed and salt-soaked	HPLC-ESI-MS/MS	Total flavonoids 114.50 mg/g vs. 113.60 mg/g	[46]
	*Astragalus membranaceus* (Fisch.) Bunge	Root	Stir-fried Salt-soaked	HPLC-ESI-MS/MS	Total flavonoids 114.50 mg/g vs. 113.70 mg/g	[46]
	*Houttuynia cordata* Thunb.	Whole plant	Natural air drying	GC–MS, HPLC	Quercitrin 0.413% vs. 0.323%	[47]
Volatile oil	*Zingiber officinale* Roscoe	rhizome and root	Sun-drying (fresh-cut slices, 10 d, indoor/outdoor, 23–25 °C, 40–45% RH)	HPLC	Volatile oil yield 0.29% vs. 0.22%, 2,6-octadienal, 3,7-dimethyl, (Z) 9.15 mg/g vs. 7.40 mg/g, 2-heptanol 0.39 mg/g vs. 0.26 mg/g, geraniol 5.71 mg/g vs. 5.35 mg/g, linalool 12.09 mg/g vs. 9.60 mg/g, borneol 2.62 mg/g vs. 0.53 mg/g	[48]
	*Houttuynia cordata* Thunb.	Whole plant	Natural air drying	GC–MS, HPLC	Volatile oil 2.63 mL/kg vs. 0.41 mL/kg	[47]
	*Curcuma phaeocaulis* Valeton	Leaves	Air-drying (shade)	GC–MS	alpha-pinene 6.47% vs. 4.17%, beta-myrcene 1.25% vs. 1.02%, Z-13,7-dimethyl-3,6-octatriene 0.68% vs. 0.18%, 2-carene 1.24% vs. 0.83%, beta-elemene 4.69% vs. 1.29%, 2-octanol 0.45% vs. 0.23%, 2-heptanol, 3-methyl 0.45% vs. 0.41%, 2-nonanone 0.67% vs. 0.33%, isoborneol 1.69% vs. 1.33%, furanodiene 9.26% vs. 6.93%, curdione 21.33% vs. 19.73%, curcumol 11.73% vs. 8.96%	[49]
	*Chrysanthemum morifolium* Ramat.	Flower heads	Hot-air drying (60 °C)	GC–MS, HPLC	Borneol 1.69% vs. 1.33%, caryophyllene oxide 2.44% vs. 2.14%, camphor 6.17% vs. 5.96%, humulene 0.68% vs. 0.48%, curcumol 11.73% vs. 9.81%, linalool 0.24% vs. 0.20%, cedrene-8-ol-13-ol 1.43% vs. 0.81%, *α*-copaene 0.72% vs. 0.60%, caryophyllene 2.14% vs. 1.82%, 2,6-octadienal,3,7-dimethyl,(Z) 9.15% vs. 7.4%, linalool 5.71% vs. 5.35%, 2,6-octadienal,3,7-dimethyl,(E) 11.7% vs. 9.81%, 7-oxabicyclohexadeca-4,1-methyl-4-(1-methylethyl). 0.29% vs. 0.27%, humulene. 0.68% vs. 0.48%	[50]
	*Panax quinquefolium* L.	Root	Sun-drying (40 °C)	GC-MS	Volatile oil content 0.11% vs. 0.04%	[51]
	*Angelica sinensis* (Oliv.) Diels	Root	Stir-frying with yellow wine	HPLC	Volatile oil 0.69% vs. 0.60%	[52]
	*Angelica sinensis* (Oliv.) Diels	Root	Stir-frying with soil powder	HPLC	Volatile oil 0.69% vs. 0.39%	[52]
	*Angelica sinensis* (Oliv.) Diels	Root	Stir-frying to carbon	HPLC	Volatile oil 0.69% vs. 0.13%	[52]
	*Cinnamomum migao* H.W.Li	Fruit	Drying	GC–MS	Camphene 0.25% vs. 0.09%, pinene 0.59% vs. 0.13%, myrcene 1.87% vs. 0.19%, citronellal 2.67% vs. 0.52%, citral 36.51% vs. 10.02%, geraniol 0.51% vs. 0.16%, *α*-citral 43.45% vs. 12.64%, (+)-(R)-citronellol 0.22% vs. 0.17%, eucalyptol 0.22% vs. 0.13%	[21]
	*Artemisia annua* L.	Above-ground parts	Air-drying in the shade	HPLC	Volatile oil 0.21% ± 0.05 vs. 0.16% ± 0.08, *α*-pinene: 6.47% vs. 4.17%, *β*-myrcene: 1.25% vs. 1.02%, tricycloheptane: 0.43% vs. -, bicycloheptane: 0.56% vs. -, (Z)-3,7-dimethyl-3,6-octatriene: 0.68% vs. 0.18%, *β*-elemene: 4.69% vs. 1.29%, humulene: 0.23% vs. -, caryophyllene oxide: 2.44% vs. 2.14%, *α*-bergamotene: 0.86% vs. -, *α*-farnesene: 0.11% vs. -, 2-octanol: 0.45% vs. 0.23%, 2-nonanone: 0.67% vs. 0.33%	[49]
	*Aloe vera* (L.) Burm. f.	Flowers	Natural drying (80–90 °C)	GC-MS	Total volatile oil content 2.81% vs. 2.23%, ethane, 1,1-diethoxy- 13.88% vs. -, 2-pentanol, formate 1.58% vs. -, benzene, 1,3-dimethyl- 3.41% vs. -, cyclohexanone, 2,3,3-trimethyl- 4.38% vs. -, 5-butyl-6-hexyloctahydroxyl- 2.19% vs. -, butylated hydroxytoluene 20.55% vs. 5.86%, cyclohexane, 1-(cyclohexylmethyl)- 1.75% vs. -, cyclohexane, 1,2,4,5-tetraethyl- 2.76% vs. -, diallyldivinylsilane 4.40% vs. -, *α*-cedrene oxide 1.12% vs. -, aciphyllyl alcohol 0.68% vs. -, bicyclo [7.7.0]hexadec-1(9)-ene 0.29% vs. -, cyclohexanone, 3-methyl-2-(1-methyl-) 0.58% vs. -, imidazole, 4-methyl-5-[3,3,3-trimethyl-] 0.71% vs. -, 3-cyclohexene-1-carboxaldehyde 0.24% vs. -	[53]
	*Cinnamomum camphora* (L.) Presl	Leaf	Air-dried	GC-MS	(±)-Cadinene 6.25% vs. 5.23%, 6-Cineole-4-ol 65.85% vs. 55.98%. Eucalyptol 6.69% vs. 8.43%	[54]
	*Cinnamomum camphora* (L.) Presl	Branch	Air-dried	GC-MS	6-carene-4-ol 41.29% vs. 32.29%	
	*Cinnamomum camphora* (L.) Presl	Root	Air-dried	GC-MS	Camphor 11.01% vs. 9.16%. Safrole 74.82% vs. 71.83%	
	*Astragalus membranaceus* (Fisch.) Bunge var. *mongholicus* (Bunge) P. K. Hsiao	Root	Air-dried to 10–13% moisture content	SED followed GC-MS	ethyl-3-buten-2-ol 0.32% vs.-, 1-penten-3-ol 0.9% vs. -, (Z)-2-penten-1-ol 1.12% vs.-, 1-Octen-3-Ol 1.06% vs. 0.55%, 1-hexanol 27.5% vs. 9.67%, (Z)-3-hexen-1-ol 0.3 vs.-, (E)-2-hexen-1-ol 10.39% vs. 2.02%, 3,7-dimethyl-1.6-octadien-3-ol 0.21% vs. 0%, (E)-2-octen-1-ol 0.16% vs.-, 1-nonen-4-Ol 0.47% vs.-, p-menth-1-en-8-ol 0.28% vs.-, (E)-2-nonen-1-ol 0.30% vs.-, (E)-2-hexenal 8.67% vs.-, 2,2,4-trimethyl-5-hexen-3-ol 1.04% vs.-, (E)-2-hexenal 8.67% vs.-, (Z)-2-heptenal 0.47% vs.-, furaldehyde 0.69% vs.-, n-caproic acid vinyl ester 10.19% vs.-, heptadecane 1.47% vs.-, octanoic acid 0.20% vs.-, heptadecane 0.20% vs.-, nonadecane 0.26% vs.-, eicosane 0.29% vs.-, heneicosane 0.38% vs.-, limonene 0.41% vs.-, 1-hexadecane 0.3 vs.-, ethylbenzene 4.99 vs.-, o-xylene 13.62 vs.-, propyl-benzene 0.51 vs.-,1-ethyl-3-methyl-benzene 2.76% vs.-, 1-ethyl-4-methyl-benzene 4.51% vs.-, 1,2,4-trimethyl-benzene 1.94% vs.-	[55]
	*Litsea glutinosa* (Lour.) C.B.Rob.	Leaves	Sun-drying	GC-MS	Phytol 6.90% vs. 4.74%, *β*-pinene 6.79% vs. 3.65%, *α*-pinene 5.97% vs. 5.42%, caryophyllene oxide 5.95% vs. 3.90%, myrcene 2.81% vs. 1.67%	[56]
Acids	*Rosmarinus officinalis* L.	Leaves	Hot-air drying (40 °C)	HPLC, Ultraviolet-visible spectroscopy	Carnosic acid 8.00% vs. 2.00%, rosmarinic acid 3.50% vs. 1.20%	[57]
	*Portulaca oleracea* L.	Whole plant	Open-air drying	Ultra-Performance Liquid Chromatography coupled with Quadrupole Time-of-Flight Mass Spectrometry	Ferulic acid 17.68 vs. 0.16 μg/g, caffeic acid 9.67 vs. 0.13 μg/g	[36]
	*Angelica sinensis* (Oliv.) Diels	Root	Stir-frying with yellow wine	HPLC	Ferulic acid 0.07% vs. 0.06%	[52]
	*Angelica sinensis* (Oliv.) Diels	Root	Stir-frying with soy powder	HPLC	Ferulic acid 0.07% vs. 0.02%	[52]
	*Angelica sinensis* (Oliv.) Diels	Root	Stir-frying to carbon	HPLC	Ferulic acid 0.07% vs. 0.01%	[52]
	*Perilla frutescens* (L.) Britton	Leaves	Dried (60 °C hot-air drying for 12 h)	HPLC	Rosmarinic acid 18,468.00 mg/g vs. 43.13 μg/g	[58]
	*Sedum aizoon* L.	Roots, stems, and leaves.	Hot-air drying (80 °C)	HPLC	Gallic acid 1.00 mg/g vs. 0.95 mg/g	[59]
Vitamins	*Panax quinquefolium* L.	Root	Sun-drying (40 °C)	Ultraviolet-visible spectroscopy	Vitamin C 0.26% vs. 0.19%, Vitamin E 0.37% vs. 0.27%	[51]
	*Melissa officinalis* L.	Leaves and shoot tops	Air-drying (25–32 °C)	Tillmans	Vitamin C 53.20 mg/100 g vs. 3.30 mg/100 g	[60]
Others	*Panax ginseng* C. A. Mey.	Roots and rhizomes	Sun-drying	Reverse-phase high-performance liquid chromatography	L-pyroglutamic acid 1.06 mg/g vs. 0.32 mg/g	[61]

Abbreviation: GC-MS, gas chromatography–mass spectrometry; HPLC, high-performance liquid chromatography; HPLC-ELSD, Ultra-Performance Liquid Chromatography coupled with Quadrupole Time-of-Flight Mass Spectrometry; “-” means not detected in the original study.

## Data Availability

No new data were created or analyzed in this study.

## Supplementary Materials

The following supporting information can be downloaded at https://www.mdpi.com/article/10.3390/plants15142122/s1.

## Abbreviations

FPs	fresh phytomedicines
DPs	dried phytomedicines
CNKI	China National Knowledge Infrastructure
HPLC	high-performance liquid chromatography
GC-MS	gas chromatography–mass spectrometry
*S. aureus*	*Staphylococcus aureus*
*E. coli*	*Escherichia coli*
*B. subtilis*	*Bacillus subtilis*
*P. vulgaris*	*Proteus vulgaris*
*S. dysenteriae*	*Shigella dysenteriae*
MIC	minimum inhibitory concentrations
DPPH	2,2-diphenyl-1-picrylhydrazyl
ABTS^+^	2,2′-azino-bis (3-ethylbenzothiazoline-6-sulfonic acid) radical cation
·OH	hydroxyl radical
TNF-α	tumor necrosis factor-α
IL-1β	interleukin-1β
