# Supplementary material for: Fresh Phytomedicines: Traditional Applications, Chemical Composition, Pharmacological Activities, Challenges, and Strategies
Source: Plants (Basel). 2026 Jul 9;15(14):2122. doi: 10.3390/plants15142122 (PMC13416491; doi:10.3390/plants15142122)
Supplement: Supplementary file 1 [file plants-15-02122-s001.zip › Supplementary Table S1.pdf]

Supplementary Table S1. Representative experimental models and studies selected from Table 3 to support the comparative analysis of fresh and dried phytomedicines shown in Fig. 6.

Pharmacological Activity	Plant Name	Test Subject	Concentration/Dosage	Fresh Plant (FPs) Activity Index	Dried Plant (DPs) Activity Index	Relative Activity (FPs/DPs, %)	Ref.
Antimicrobial activity	*Thymus pulegioide* L.	Alternaria brassicae	-	Diameter of Inhibition Zone 25.30 ± 0.29 mm	Diameter of Inhibition Zone 21.71±0.17 mm	116.5	[1]
Antidiabetic activity	*Geranium divaricatu* Ehrh.	High-fat diet/streptozotocin-induced type 2 diabetic mice	80 g/kg/day, intragastric administration for 15 days	Fasting blood glucose decreased from 17.48 mmol/L to 8.85 mmol/L (reduction rate 49.4%)	Fasting blood glucose decreased from 17.48 mmol/L to 12.17 mmol/L (reduction rate 30.3%)	163.00	[2]
Antidiabetic activity	*Portulaca oleracea* L.	Streptozotocin-induced C57BL/6J diabetic mice	400 mg/kg/day, intragastric administration for 3 weeks	Fasting blood glucose was reduced by 31.46%	Fasting blood glucose reduced by 14.0%	224.70	[3]
Immunoregulatory activity	*Rehmannia glutinosa* (Gaertn.) Libosch. ex Fisch. & C.A.Mey.	Peritoneal macrophage phagocytosis rate test	10 g/kg, intragastric administration	Peritoneal macrophage phagocytosis rate 38.6%	Peritoneal macrophage phagocytosis rate 26.4%	146.20	[4]
Antioxidant activity	*Dendrobium officinale* Kimura & Migo	$ABTS^+$ free radical scavenging assay	4.00 mg/mL	$ABTS^+$ free radical scavenging rate 61.24%	$ABTS^+$ free radical scavenging rate 48.01%	127.60	[5]
Antitumor activity	*Typhonium giganteum* Engl.	Tumour-bearing mouse model	0.20 mL/10 g	Tumour inhibition rate 47.6%	Tumour inhibition rate 34.8%	136.80	[6]
Anti-inflammatory activity	*Perilla frutescens* (L.) Britton	NO production inhibition test (LPS-induced inflammation model)	100 μg/mL	NO production inhibition rate 0.47 (relative value)	NO production inhibition rate 0.12 (relative value)	391.70	[7]
Antiemetic activity	*Zingiber officinale* Roscoe	Pigeons induced by $CuSO_4$.	10.0 g/kg (crude drug), intragastric administration for 3 days	Antiemetic rate 89.36%	Antiemetic rate 61.30%	146.00	[8]
Neuroprotective activity	*Panax ginseng* C.A.Mey.	PC12 cell injury model	1 00 mg /mL	Cell viability 94.47%	82.97%	114.00	[9]
Antivenom	*Sedum sarmentosum* Bunge	Snakebite-induced oedema model	Reduction of local swelling	The survival rate of mice is 33.3%	The survival rate of mice is 16.7%	199.4	[10]

Note: The studies included in this table were selected from Table 3 based on the availability of direct comparisons between fresh and dried phytomedicines derived from the same botanical source and evaluated under identical extraction procedures, dosing regimens, and experimental conditions. These representative studies were used to illustrate the comparative trends summarised in Fig. 8

References:

1. Chen, P. P., Ma, X. J., Liu, L., Wu, H., Lin, J. X., Lan, M. B., Zhuang, W. D. Component analysis and comparison of antibacterial activity of essential oil from fresh and dry leaves and stems of *Thymus pulegioides*. *Subtropical Plant Sci.* **2021**, 50,1,15-20.
2. Wei, N. Q., Liu, J. H., Wei, M. M., Li, Q., Sun, J., Xue, Z. F., Li, L. Comparative study of fresh and dry *Gynura divaricata* (L.) DC. on improving glucose and lipid metabolism, oxidative stress and inflammation in diabetes mice. *J. Chin. Med. Mater.* **2020**, 43,12,3040-3044.https://doi.org/10.13863/j.issn1001-4454.2020.12.036
3. Gu, J F, Zheng, Z Y, Yuan, J R, Zhao, B J, Wang, C F, Zhang, L, Xu, Q Y, Yin, G W, Feng, L, Jia, X B. Comparison on hypoglycemic and antioxidant activities of the fresh and dried *Portulaca oleracea* L. in insulin-resistant HepG2 cells and streptozotocin-induced C57BL/6J diabetic mice. *J. Ethnopharmacol.* **2014**, 161,214-223.https://doi.org/
4. Yi, H., Liang, A. H., Xue, B. Y., Wang, J. H., Hao, J. D. Comparative study on the hemostatic and immune effects of fresh and dried *Rehmannia glutinosa*. *J. Tradit. Chin. Med.* **1999**, 24,11,663-666.
5. Zhang, Y. T. Study on the differences of polysaccharide components and antioxidant activities in fresh and dry D*endrodium officinale* Kimura et migo. Anhui: Anhui University of Chinese Medicine, **2017**.
6. Zhou, L., Jia, J., Liu, Y. H. Effect of giant *Typhonium giganteum* Engl. on antitumor activity in mice before and after processing. *Chin. J. Cancer Prev. Treat.* **2018**, 25,3-5.
7. Tantipaiboonwong, P, Pintha, K, Chaiwangyen, W, Suttajit, M, Khanaree, C, Khantamat, O. Bioefficacy of nga-mon (*Perilla frutescens*) fresh and dry leaf: assessment of antioxidant, antimutagenicity, and anti-inflammatory potential. *Plants* **2023**, 12,11, 2210.https://doi.org/10.3390/plants12112210
8. Wang, J. H., Xue, B. Y., Liang, A. H., Wang, L., Hao, J. D. Comparative study on the pharmacological activities of *Zingiber officinale* Roscoe and dried *Zingiber officinale* Roscoe. *Chin. Pharm. J.* **2000**, 35,3,21-23.
9. Jiang, Y. M., Li, Z. Y., Liu, X. M., Chang, Q., Zhang, J., Pan, R. L. Comparative study on the neuroprotective effect of dry and fresh *Panax ginseng* C.A.Mey. against the damage in PC12 cells. *Nat. Prod. Res. Dev.* **2014**, 26,7,1072-1076, 1055.https://doi.org/10.16333/j.1001-6880.2014.07.021
10. Ren, Y. S., Zhang, T. P., Liang, S., Li, Y. Q., Mei, Z. N., Liao, M. C. The efficacy superiority of fresh *Sedum sarmentosum* on snakebite treatment. *J. South-Central Univ. Natl.* **2020**, 39,2,151-156.
